# SCAMP3 is a component of the *Salmonella*-induced tubular network and reveals an interaction between bacterial effectors and post-Golgi trafficking

**DOI:** 10.1111/j.1462-5822.2009.01329.x

**Published:** 2009-06-03

**Authors:** Luís Jaime Mota, Amy E Ramsden, Mei Liu, J David Castle, David W Holden

**Affiliations:** 1 Centre for Molecular Microbiology and Infection, Imperial College LondonArmstrong Road, London SW7 2AZ, UK.; 2 Department of Cell Biology and Cell and Developmental Biology Program, University of VirginiaCharlottesville, VA 22908, USA.

## Abstract

*Salmonella enterica* are facultative intracellular bacterial pathogens that proliferate within host cells in a membrane-bounded compartment, the *Salmonella*-containing vacuole (SCV). Intracellular replication of *Salmonella* is mediated by bacterial effectors translocated on to the cytoplasmic face of the SCV membrane by a type III secretion system. Some of these effectors manipulate the host endocytic pathway, resulting in the formation in epithelial cells of tubules enriched in late endosomal markers, known as *Salmonella*-induced filaments (SIFs). However, much less is known about possible interference of *Salmonella* with the secretory pathway. Here, a small-interference RNA screen revealed that secretory carrier membrane proteins (SCAMPs) 2 and 3 contribute to the maintenance of SCVs in the Golgi region of HeLa cells. This is likely to reflect a function of SCAMPs in vacuolar membrane dynamics. Moreover, SCAMP3, which accumulates on the *trans*-Golgi network in uninfected cells, marked tubules induced by *Salmonella* effectors that overlapped with SIFs but which also comprised distinct tubules lacking late endosomal proteins. We propose that SCAMP3 tubules reflect a manipulation of specific post-Golgi trafficking that might allow *Salmonella* to acquire nutrients and membrane, or to control host immune responses.

## Introduction

*Salmonella enterica* serovar Typhimurium (*S.* Typhimurium) causes gastroenteritis in humans and is commonly used to study the molecular and cellular basis of *Salmonella* virulence. *S. enterica* invades and replicates within many host cell types. Intracellular bacteria multiply in a membrane-bounded compartment, the *Salmonella*-containing vacuole (SCV). Intravacuolar replication of *Salmonella* requires several virulence proteins, in particular those encoded by the *Salmonella* pathogenicity island (SPI)-2 type III secretion system (T3SS). The SPI-2 T3SS translocates over 20 effector proteins into the vacuolar membrane and host cell cytoplasm (Haraga *et al*., 2008).

Like other vacuolated pathogens (Brumell and Scidmore, 2007), the capacity of *Salmonella* to survive and multiply intracellularly is dependent on interactions with host cell vesicular trafficking pathways. For years, *Salmonella* has been regarded as a paradigm of a pathogen that interacts selectively with the host endocytic system. Numerous studies using an epithelial cell infection model have described SCV maturation in terms of sequential interactions with the endocytic pathway and the physical displacement of bacterial vacuoles towards the microtubule-organizing center (MTOC) (Steele-Mortimer, 2008). The mature SCV is enriched in late endocytic proteins, such as LAMP1, which accumulate on the vacuolar membrane during intracellular growth of the bacteria. In epithelial cells, a striking feature of the interaction between *S.* Typhimurium and late endocytic compartments is the appearance of long tubules, known as *Salmonella*-induced filaments (SIFs), which contain a variety of late endosomal markers, including lysosomal glycoproteins such as LAMPs, vacuolar vATPase (vATPase), Niemann-Pick C1 protein and lysobisphosphatidic acid (Garcia-del Portillo *et al*., 1993; Drecktrah *et al*., 2008; Rajashekar *et al*., 2008; Steele-Mortimer, 2008). SIFs appear 4–5 h after bacterial uptake, concomitant with the onset of bacterial replication and of SPI-2 activity, and several SPI-2 effectors are involved in their formation (Ramsden *et al*., 2007a; Haraga *et al*., 2008; Steele-Mortimer, 2008).

In recent years, it has been suggested that SCVs might also interact with the host secretory pathway. This is because in epithelial cells the majority of vacuoles containing wild-type (wt) *S.* Typhimurium are maintained at the MTOC-Golgi region (Salcedo and Holden, 2003, Ramsden *et al*., 2007b), and disruption of the Golgi network with brefeldin A (BFA) inhibits the intracellular growth of the bacteria in HeLa cells and in macrophages (Salcedo and Holden, 2003). SCV positioning is dependent on the SPI-2 effectors SifA, SseF and SseG, and has been correlated with the intracellular replication of *Salmonella* (Salcedo and Holden, 2003; Boucrot *et al*., 2005; Abrahams *et al*., 2006; Deiwick *et al*., 2006; Ramsden *et al*., 2007b). In further support of the idea that SCVs might interact with the secretory pathway, experiments using the vesicular stomatitis virus glycoprotein (VSVG) or fluorescently labelled C5-ceramide indicated that transport from the Golgi to the plasma membrane is affected by SPI-2 effectors (Kuhle *et al*., 2006). However, vesicles containing these markers did not appear to fuse significantly with SCVs or with SIFs (Kuhle *et al*., 2006).

*Salmonella* effectors might determine SCV positioning by controlling cytoskeleton-based motors, or by tethering of SCVs to Golgi-associated molecules (Boucrot *et al*., 2005; Abrahams *et al*., 2006; Henry *et al*., 2006; Ramsden *et al*., 2007b; Wasylnka *et al*., 2008), but the exact mechanism is unclear. To identify host cell protein(s) that regulate SCV positioning, we undertook a small-interference RNA (siRNA) screen of proteins with functions related to the secretory pathway. We found that depletion of secretory carrier membrane proteins (SCAMPs) 2 and 3 caused the scattering of SCVs throughout the cell, and this could be suppressed by inhibiting the microtubule centrifugal motor, kinesin-1. Further experiments revealed that SCAMP3, which mainly localizes to the *trans*-Golgi network (TGN) of uninfected cells, is a major component of the tubular network induced by SPI-2 effectors that includes SIFs and also novel *Salmonella*-induced tubules lacking late endosomal markers. This establishes that SIFs contain both endosomal and secretory pathway-derived molecules, and that SCVs interact with post-Golgi trafficking pathways. The work also highlights a function of SCAMP3 in the intracellular transport of membranes.

## Results

### SCAMPs 2 and 3 contribute to the positioning of SCVs in HeLa cells

A siRNA screen in HeLa cells suggested that SCAMPs might control positioning of SCVs in the Golgi region (data not shown). SCAMPs are a family of integral membrane proteins found in animals and plants (Fernandez-Chacon and Sudhof, 2000; Hubbard *et al*., 2000), and are thought to play a fundamental role in membrane trafficking (Castle and Castle, 2005; Liao *et al*., 2008). SCAMPs 1–4 are ubiquitously expressed in the vast majority of mammalian cells and SCAMP5 is mainly expressed in neuronal cells (Fernandez-Chacon and Sudhof, 2000; Singleton *et al*., 1997).

**Fig. 1:**
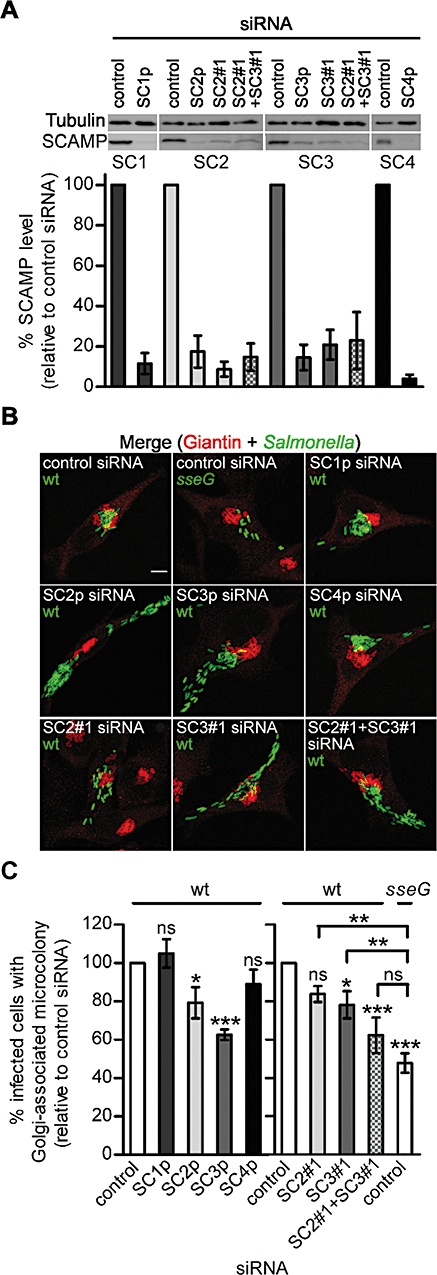
Depletion of SCAMPs affects SCV positioning. A. HeLa cells were transfected with the indicated siRNAs. Whole-cell extracts of the transfected cells were analysed by immunoblotting using anti-tubulin and isoform-specific anti-SCAMP antibodies. The intensity of the signal of the SCAMP bands was quantified by densitometry using ImageJ (NIH, USA) and normalized to the intensity of the corresponding tubulin band. The immunoblot shown is representative of five independent experiments whose quantification is displayed in the graph below. B. HeLa cells were transfected with the indicated siRNAs and infected for 14 h with wt or *sseG* mutant *S.* Typhimurium expressing GFP. The infected cells were fixed and immunolabelled for giantin. Scale bar, 5 μm. C. Quantification of SCV positioning phenotypes in cells infected as in (B). Data are from the same five independent experiments represented in (A). In these experiments 69 ± 2% of cells transfected with control siRNA and infected with wt *S.* Typhimurium showed a Golgi-associated microcolony. *P*-values were obtained by one-way anova and Dunett *post hoc* analyses (*P < 0.05; **P < 0.01; ***P < 0.001; ns – not significant) relative to control siRNA-treated cells infected with wt *S.* Typhimurium (*P*-value above bars) or *sseG* mutant bacteria (as indicated). All values are mean ± SEM (*n* = 5).

We analysed if SCAMPs 1–4 influence positioning of SCVs. HeLa cells were individually transfected with either a non-targeting control siRNA or pools (SC1p, SC2p, SC3p, SC4p), each containing four different siRNAs for each SCAMP; these pools efficiently depleted the cellular levels of the SCAMPs (Fig. 1A). SCAMP-depleted cells were then infected with wt *S.* Typhimurium constitutively expressing the green fluorescent protein (GFP). The cells were fixed 14 h post invasion (p.i.), immunolabelled for the Golgi with an anti-giantin antibody, and analysed for the presence of a bacterial microcolony within the Golgi region (see *Experimental procedures*). Knock-down of SCAMP2 or SCAMP3, but not of SCAMP1 or SCAMP4, resulted in a significant decrease in the frequency with which SCVs became clustered and centrally located (Fig. 1B and C). Next, we identified the single siRNAs within SC2p and SC3p that resulted in the more pronounced reduction in the cellular levels of SCAMPs 2 and 3 (Fig. S1A). Depletion of SCAMP2 or SCAMP3 with these single siRNAs (SC2#1 and SC3#1) essentially recapitulated the effect observed using the pools of siRNAs (Fig. 1), but the SCV positioning defect observed after knock-down of SCAMP2 was not statistically significant (Fig. 1C). However, we could further confirm that depletion of SCAMP2 or SCAMP3 affected SCV positioning by using additional single siRNAs (Fig. S1). Depletion of SCAMP2 did not affect the cellular levels of SCAMP3, or vice-versa (data not shown).

Simultaneous depletion of SCAMPs 2 and 3 (Fig. 1A) resulted in an SCV positioning phenotype, which was more pronounced than that observed after the single knock-down of each SCAMP and which was indistinguishable from that observed in cells transfected with control siRNA and infected with *sseG* mutant bacteria (Fig. 1C). Knock-down of SCAMPs 2 and 3 in HeLa cells did not noticeably affect the intracellular replication of *Salmonella*, SCV migration to the MTOC, SIF formation or the stability of the vacuolar membrane enclosing wt bacteria (Fig. S2 and data not shown). These data show that SCAMPs 2 and 3 contribute to the maintenance of SCVs in the Golgi region of HeLa cells.

### The cellular distribution of SCAMP3 is altered in *Salmonella*-infected cells

**Fig. 2:**
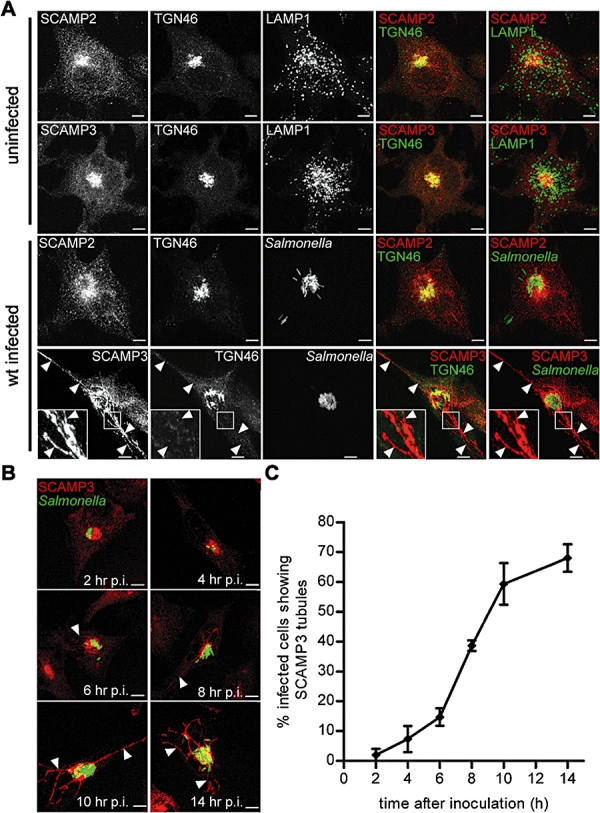
Analysis of the cellular distribution of SCAMP2 and SCAMP3. A. HeLa cells were infected with wt *S.* Typhimurium expressing GFP (or left uninfected), fixed at 14 h p.i., and immunolabelled as indicated. TGN46 is false coloured. Arrowheads indicate the position of SCAMP3 tubules. B. The infected cells were fixed at the indicated times and immunolabelled to SCAMP3. Arrowheads indicate SCAMP3 tubules. C. Quantification of the appearance of SCAMP3 tubules in cells infected as in (B). Values are mean ± SEM (*n* = 3). All scale bars, 5 μm.

We next analysed the cellular distribution of SCAMPs 2 and 3 in HeLa cells by laser scanning confocal microscopy (LSCM) after immunolabelling with several antibodies. In uninfected cells, and as expected (Singleton *et al*., 1997; Castle and Castle, 2005), the SCAMPs were concentrated in a juxtanuclear region (Fig. 2A). Both SCAMPs, and in particular SCAMP3, colocalized extensively with a TGN marker, TGN46 (Fig. 2A and Fig. S3). No significant alteration in the cellular distribution of SCAMP2 was observed at 6, 8, 10 h, or at 14 h following infection by *Salmonella* (Fig. 2A and data not shown). However, infection with wt *S.* Typhimurium caused the frequent appearance of distinct structures marked by SCAMP3 that often extended to the periphery of the cell (Fig. 2). As SCAMPs are integral membrane proteins we concluded that these structures are tubules. SCAMP3 tubules were observed from 6 h p.i. and their frequency of appearance increased dramatically until 10 h p.i., but only slightly thereafter (Fig. 2B and C). The SCAMP3 tubules that appeared at 6–8 h p.i. were typically thinner than those observed at later times (Fig. 2B). These structures were not observed in uninfected HeLa cells (Fig. 2A). In *Salmonella*-infected cells the Golgi and TGN often appear distorted (Salcedo and Holden, 2003). Similarly, labelling of TGN46 revealed a morphologically distorted TGN in many infected cells, but this protein was not present on SCAMP3 tubules (Fig. 2A). These tubular structures were not observed in infected cells after immunolabelling of giantin (a *cis*-Golgi marker), of early endosomal antigen 1, or of the transferrin receptor [TfR; a marker of the endocytic recycling compartment (ERC)] in infected cells (data not shown).

To confirm that *Salmonella* alters the cellular localization of a protein that normally resides in the TGN, we examined the distribution of SCAMP3 in uninfected HeLa cells after treatment with BFA, which causes collapse of *cis*-Golgi stacks and tubulation of the TGN and of endosomal compartments (Lippincott-Schwartz *et al*., 1989; 1991). We observed an alteration in the cellular distribution of SCAMP3 that was similar, but not identical to, BFA-dependent TGN46 redistribution (Fig. S3). However, the redistribution of SCAMP3 was clearly different to the collapse of the *cis*-Golgi and from the rapid tubulation of TfR-marked endocytic compartments observed in the same cells (Fig. S3). Altogether, these data show that the majority of SCAMP3 is present in the TGN in uninfected cells, as previously reported (Castle and Castle, 2005), and is a major component of tubules that appear in cells infected with *S.* Typhimurium from 6 to 8 h p.i.

### SCAMP3 tubules and SIFs

**Fig. 3:**
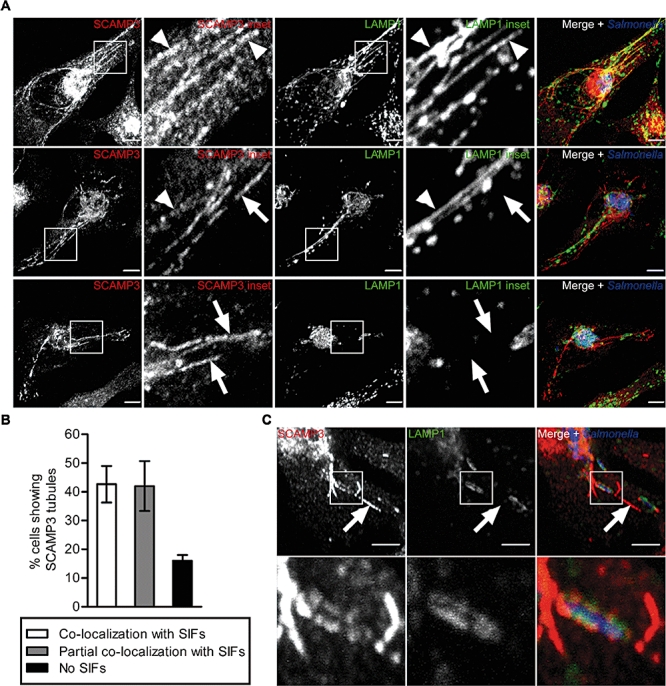
Relationship between SCAMP3 tubules and LAMP1-labelled SIFs. HeLa cells were infected with wt *S.* Typhimurium fixed at 14 h p.i., and immunolabelled as indicated. A. The images illustrate the distinction of SCAMP3 tubules from LAMP1-labelled SIFs (indicated by insets and arrows in the middle and lower panels) and also the overlap between them (indicated by insets and arrowheads in the upper and middle panels). B. Quantification of the percentage of infected cells showing SCAMP3 tubules that display SCAMP3 tubular structures colocalizing with SIFs (as exemplified in Fig. 3A, upper panel), partially colocalizing with SIFs (as illustrated in Fig. 3A, middle panel), or that do not colocalize with SIFs (see example in Fig. 3A, lower panel). Values are mean ± SEM (*n* = 3). C. HeLa cells infected with wt *S.* Typhimurium expressing GFP, fixed at 14 h p.i. and immunolabelled as indicated. LAMP1 and *Salmonella* are false coloured. The inset illustrates an example in which endogenous SCAMP3 can be detected on the SCV membrane. The arrow indicates a SCAMP3 tubule not labelled with LAMP1 (which we named SIST; see main text and Fig. 4 legend). Scale bars, 5 μm.

**Fig. 8:**
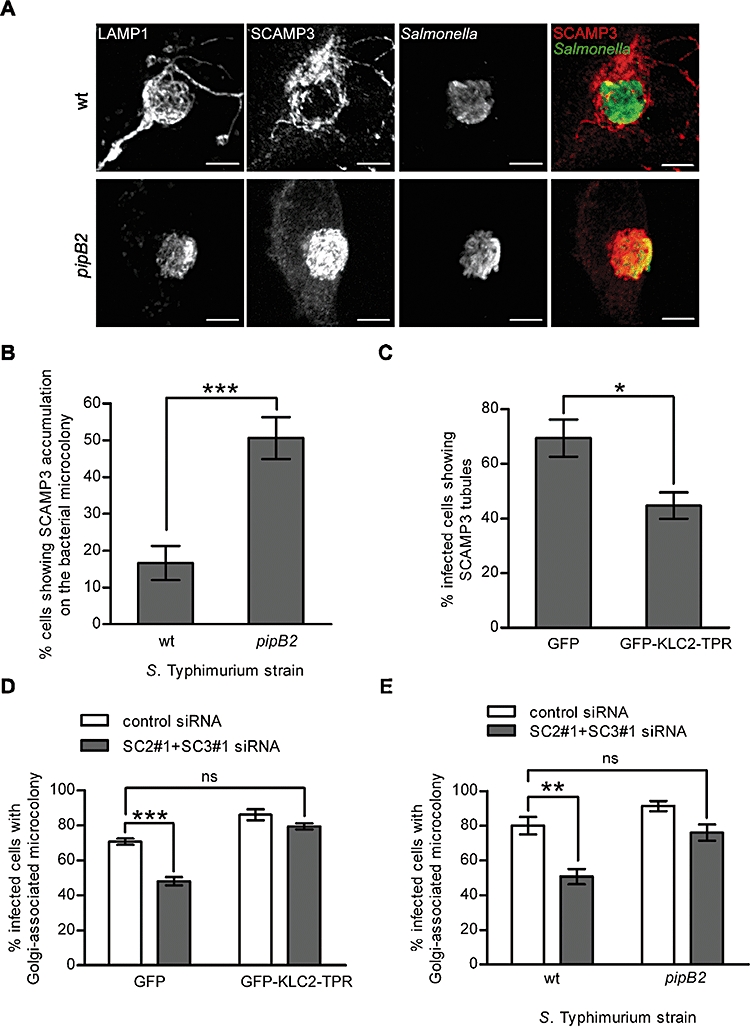
Involvement of SCAMPs 2 and 3 in the kinesin-1-driven trafficking of membranes from the SCV. HeLa cells were infected with the indicated *S.* Typhimurium strains, fixed at 14 h p.i. and immunolabelled as shown. Scale bars, 5 μm. B. Quantification of the accumulation of SCAMP3 within the microcolony in cells infected and immunolabelled as in (A). C. HeLa cells were transfected with pEGFP-C1 or with pGFP-KLC2-TPR and then infected with wt *S.* Typhimurium for 14 h. After fixation, the cells were immunolabelled for SCAMP3 and *Salmonella* and the appearance of SCAMP3 tubules was quantified. D. HeLa cells were transfected with the indicated siRNAs and then transfected and infected as explained in (C). The cells were immunolabelled for giantin and *Salmonella* and the SCV positioning phenotype was quantified. E. HeLa cells were siRNA-transfected as shown, infected with the indicated *S.* Typhimurium strains expressing GFP, fixed at 14 h p.i. and immunolabelled to giantin. The SCV positioning phenotype was quantified. The *P*-values were obtained by one-way anova and Dunett *post hoc* analyses (D and E) or a two-tailed unpaired Student's *t*-test (B and C) (*P < 0.05; **P < 0.01; ***P < 0.001; ns, not significant). All values are mean ± SEM (*n* = 3).

To assess if SCAMP3 tubules represent SIFs, we infected HeLa cells for 14 h with wt *S.* Typhimurium. Cells were then immunolabelled for SCAMP3, LAMP1 and *Salmonella*. In 43 ± 6% of cells displaying SCAMP3 tubules, the SCAMP3 structures were colabelled with continuous or patched LAMP1-labelling along part of their length (Fig. 3A, upper panel, and 3B); in 41 ± 8% of cells, at least one SCAMP3 tubule did not colocalize with LAMP1 (Fig. 3A, middle panel, and 3B), and in 16 ± 2% of cases, no SIFs were evident (Fig. 3A, lower panel, and 3B). The appearance of SIFs with no detectable SCAMP3 was very rare. Because SIFs are an extension of the SCV, we used LSCM to analyse if endogenous SCAMP3 also localizes to the vacuolar membrane. SCAMP3 encircling wt bacteria was only detected on rare occasions (Fig. 3C), and in these cases the protein followed a labelling pattern similar to LAMP1 (Fig. 3C). Moreover, SCAMP3 did not normally accumulate around the bacterial microcolony (see Fig. 8 below).

**Fig. 4:**
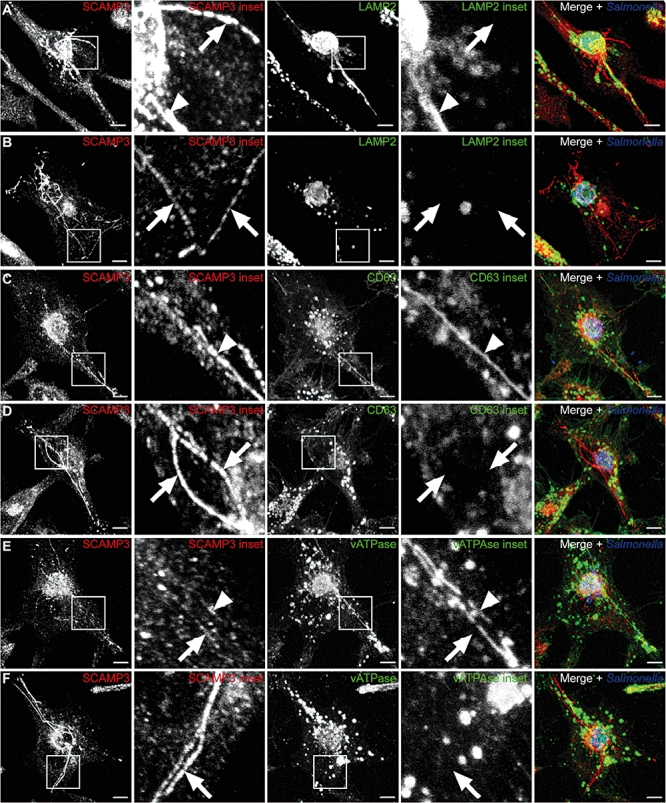
Relationship between SCAMP3 tubules and SIFs labelled with various late endosomal markers. HeLa cells were infected with wt *S.* Typhimurium expressing GFP, fixed at 14 h p.i., and immunolabelled as indicated. The images illustrate that SCAMP3 is present on SIFs labelled with LAMP2, CD63 or vATPase (indicated by arrowheads in A, C and E), but that SCAMP3 tubules can also be found that do not contain late endosomal markers (indicated by arrows in A, B, D, E, and F; and see also Figs 3 and 5). We named these *S**almonella-*induced SCAMP3 tubules lacking endosomal markers as SISTs. All scale bars, 5 μm.

To test further if SCAMP3-marked tubules could be distinguished from SIFs, HeLa cells infected for 14 h with wt *S.* Typhimurium expressing GFP were immunolabelled for SCAMP3 and for the late endosomal proteins LAMP2, LAMP3 (CD63) and vATPase. Tubular structures containing SCAMP3 but not LAMP2 were found both in infected cells showing clear LAMP2-marked SIFs (Fig. 4A) and in infected cells not displaying SIFs (Fig. 4, panel B). Labelling of CD63 or vATPase also revealed SIFs containing SCAMP3 in infected cells (Fig. 4C and E), and SCAMP3 tubules not labelled for those proteins were also evident (Fig. 4D–F). Therefore, SCAMP3-marked tubules that did not contain detectable levels of LAMP1, LAMP2, CD63 or vATPase could be readily identified in many infected cells at 14 h p.i. (Figs 3 and 4). Furthermore, SCAMP3 was also present in SIFs labelled with those late endocytic proteins (Figs 3 and 4). SCAMP3 tubules could also be distinguished from LAMP1-labelled SIFs at 8, 10 and 12 h p.i. (Fig. S4). However, at 8 and 10 h p.i. the majority of tubular SCAMP3 colocalized with LAMP1 on SIFs (Fig. S4). We refer to the *S**almonella-*induced SCAMP3 tubules lacking late endosomal markers as SISTs, and propose that tubules containing late endosomal markers with or without SCAMP3 continue to be referred to as SIFs. We define the entire tubular network that contains SCAMP3 (including SISTs and SIFs) as SCAMP3 tubules.

Because several SPI-2 effectors have been shown to localize to SIFs and to the SCV membrane (Ramsden *et al*., 2007a), we quantified the localization of the SPI-2 effector SseF to SCAMP3 tubules. We infected HeLa cells with *sseF* mutant bacteria harbouring a plasmid expressing SseF, C-terminally tagged with the influenza virus hemagglutinin epitope (SseF-HA). In 96 ± 2% of infected cells containing SCAMP3 tubules, these structures colocalized with SseF-HA labelling (Fig. S5). SISTs (tubules not containing LAMP1) colocalizing with SseF-HA were also evident (Fig. S5, middle and lower panels). Other bacterially translocated HA-tagged SPI-2 effectors (PipB, PipB2, SifA, SseG, SseJ) were also present on SCAMP3 tubules (data not shown).

### SISTs and SIFs in living cells

**Fig. 5:**
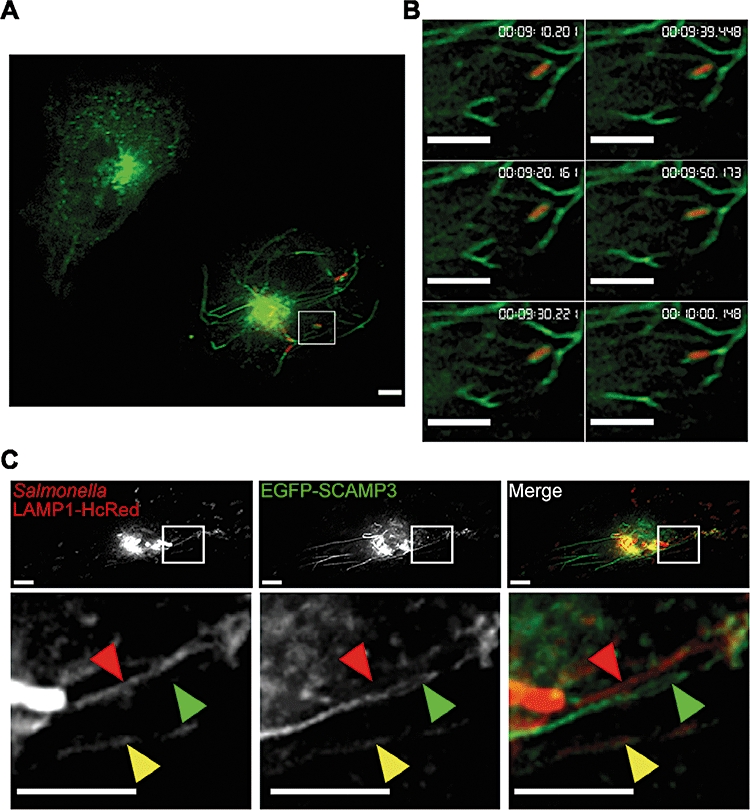
Live-cell imaging of SCAMP3 tubules and SIFs. A. Still image from Movie S1. HeLa cells were transiently transfected with pEGFP-SCAMP3 and infected with wt *S.* Typhimurium expressing DsRed. The image shows one uninfected cell (upper left corner) and one infected cell (lower right corner). The box indicates a region of the cell 4× magnified in (B). B. Last six consecutive still images from Movie S4. Note the dynamics of EGFP-SCAMP3 surrounding the bacteria. C. Still image from Movie S2. HeLa cells were transiently transfected with pEGFP-SCAMP3 and with pLAMP1-HcRed and infected with wt *S.* Typhimurium expressing DsRed. The lower panel is a 6× magnification of the inset. The red arrowhead indicates a SIF having nearly undetectable EGFP-SCAMP3; the green arrowhead indicates a SCAMP3 tubule with no detectable LAMP1-HcRed (a SIST); and the yellow arrowhead indicates a SIF containing SCAMP3. All scale bars, 5 μm.

To ascertain that the results described above were not an artifact of fixation or due to differential sensitivity of labelling, we next imaged SCAMP3 tubules in living cells by time-lapse fluorescence microscopy. HeLa cells were transfected with a plasmid encoding human SCAMP3 fused at its N-terminus with enhanced GFP (pEGFP-SCAMP3) and then infected with wt *S.* Typhimurium expressing DsRed. In uninfected cells, EGFP-SCAMP3 displayed a juxtanuclear localization similar to endogenous SCAMP3 (Fig. 5A and Movie S1). In infected cells, EGFP-SCAMP3 was reorganized into a dramatic network of SCAMP3 tubules that appeared as both dynamic and stable structures (Fig. 5A and Movie S1). Interestingly, in uninfected HeLa cells EGFP-SCAMP3 was also found on short-lived tubules (Movie S1). To simultaneously image SCAMP3 tubules and SIFs, we transfected HeLa cells with pEGFP-SCAMP3 and with a plasmid encoding LAMP1 fused at its C-terminus to HcRed (pLAMP1-HcRed). After *Salmonella* infection, SISTs (SCAMP3 tubules with no detectable LAMP1-HcRed), SIFs containing EGFP-SCAMP3 and SIFs with little EGFP-SCAMP3 were observed within the same cell (Fig. 5C and Movies S2 and S3). This confirms that SISTs are distinct from SIFs and that SCAMP3 is also present on SIFs. Furthermore, EGFP-SCAMP3 was clearly present around wt bacteria expressing DsRed, and concentrated at regions from which SCAMP3 tubules appeared to emanate (Fig. 5B and Movie S4). Because endogenous SCAMP3 was only infrequently found on the SCV membrane (see above; Fig. 3C), these observations suggest that SCAMP3 is transiently recruited to the vacuolar membrane surrounding wt bacteria, but that the levels of the endogenous protein on the bacterial vacuolar membrane at steady state are low.

Altogether, these experiments show that the tubular network extending from SCVs includes not only late endocytic proteins and SPI-2 effectors but also SCAMP3, which mainly localizes to the TGN in uninfected cells. Moreover, SISTs are devoid of late endosomal proteins, and represent a significant proportion of the *Salmonella*-induced tubular network.

### SIST formation requires the secretory pathway

**Fig. 6:**
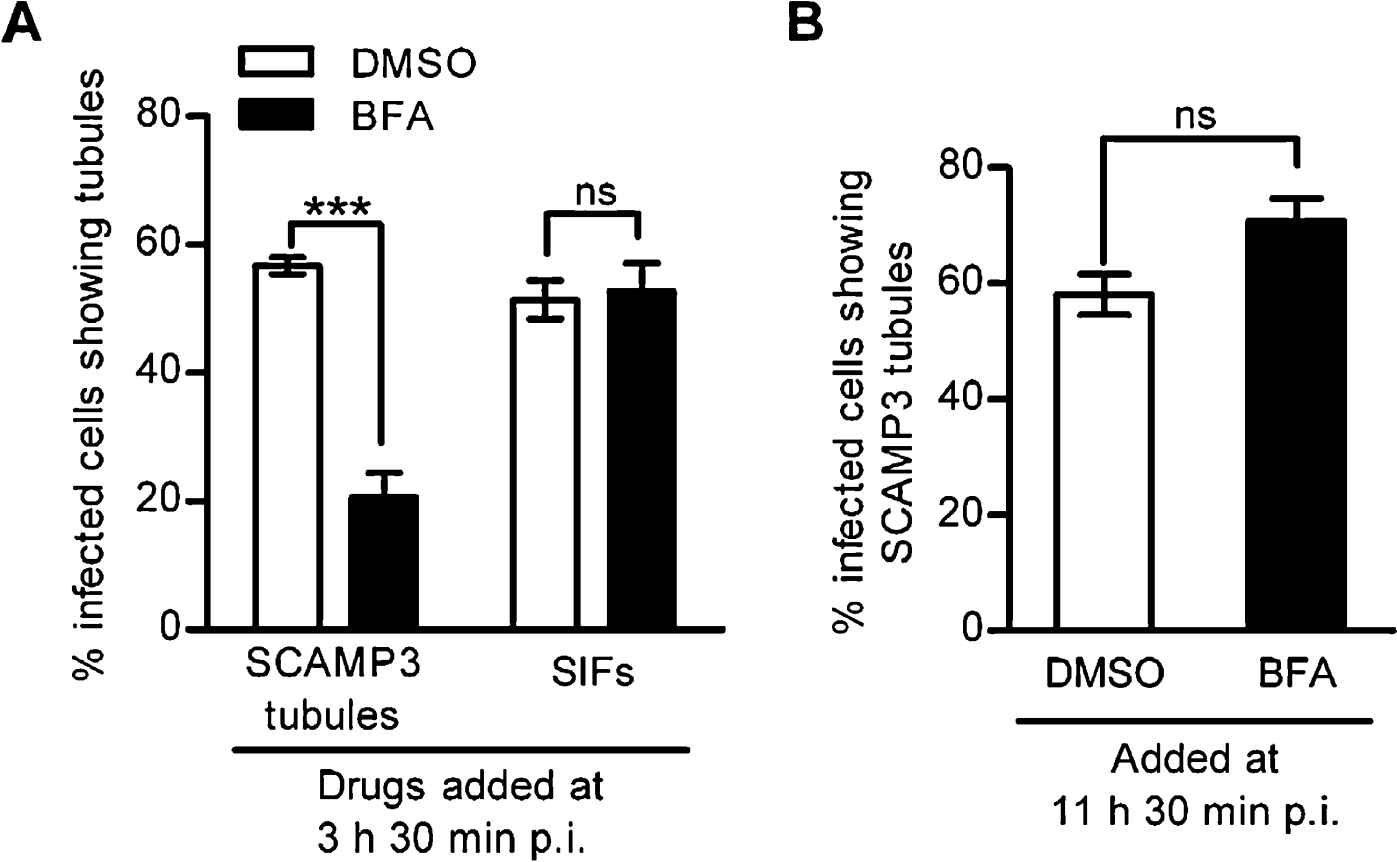
Role of the host cell secretory pathway in the formation of SCAMP3 tubules. A. Quantification of the effect of BFA treatments SCAMP3 tubulation and SIF formation. HeLa cells were infected with wt *S.* Typhimurium expressing GFP, treated with BFA or the DMSO solvent control at 3 h 30 min p.i., fixed at 12 h p.i. and immunolabelled to SCAMP3. B. Quantification of the effect of BFA in the integrity of SCAMP3 tubules. HeLa cells were infected and immunolabelled as in (A), and the drug treatment was as indicated. The *P*-values were obtained a two-tailed unpaired Student's *t*-test (***P < 0.001; ns – not significant). All values are mean ± SEM (*n* = 3).

Consistent with the late endosomal origin of SIFs, their biogenesis is not affected by disruption of the secretory pathway (Brumell *et al*., 2001). To further compare SISTs with SIFs, we analysed the effect of BFA (to disrupt the secretory pathway) on *Salmonella*-induced SCAMP3 tubulation. HeLa cells were infected with wt *S.* Typhimurium expressing GFP, and BFA was added at 3 h 30 min p.i. (prior to the first appearance of SCAMP3 tubules). The cells were fixed at 12 h p.i., immunolabelled for SCAMP3 or LAMP1, and analysed in parallel for SCAMP3 tubulation or SIFs respectively. Incubation of the cells with BFA had no effect on the frequency of appearance of SIFs but strongly inhibited SCAMP3 tubulation (Fig. 6A). This confirms that, as previously reported (Brumell *et al*., 2001), SIF formation does not require the secretory pathway and shows that SCAMP3 tubulation, and therefore SIST formation, does.

We next determined if the integrity of SCAMP3 tubules was affected by exposure to BFA. HeLa cells were infected with wt *S.* Typhimurium expressing GFP, BFA was added at 11 h 30 min p.i., and the cells were fixed at 12 h p.i. Although SCAMP3 normally localizes to the TGN and undergoes tubulation and dispersion following BFA treatment (Fig. S3), the integrity of SCAMP3 tubules was not affected by BFA (Fig. 6B). Because the TGN is equally affected by BFA in uninfected cells and in *Salmonella*-infected cells (data not shown), this indicates that SCAMP3 tubules become functionally as well as morphologically distinct from the TGN.

### Tubulation of SCAMP3 requires SPI-2 effectors

**Fig. 7:**
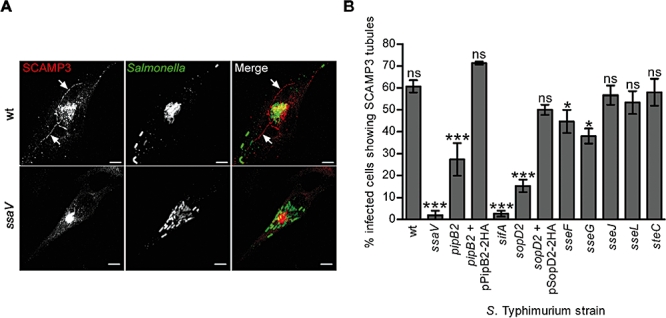
Role of the SPI-2 T3SS in SCAMP3 tubulation. A. HeLa cells were infected with the indicated *S.* Typhimurium strains for 14 h, fixed and immunolabelled to SCAMP3 and to *Salmonella*. SCAMP3 tubules are indicated by arrows. Scale bars, 5 μm. B. Quantification of the frequency at which infection of HeLa cells with the indicated *S.* Typhimurium strains results in the appearance of SCAMP3 tubules. The *P*-values were obtained by one-way anova and Dunett *post hoc* analyses (*P < 0.05; ***P < 0.001; ns, not significant). All values are mean ± SEM (*n* = 3).

To determine if tubulation of SCAMP3 requires the SPI-2 T3SS, HeLa cells were infected with wt *S.* Typhimurium or with an *ssaV* mutant (deficient for the SPI-2 T3SS machinery), for 14 h. A functional SPI-2 T3SS was essential for the appearance of SCAMP3 tubules (Fig. 7A and B). We then investigated whether SPI-2 effectors involved in SIF formation and, more generally, in modulation of host cell membrane trafficking are required for SCAMP3 tubulation. HeLa cells were infected for 14 h with wt *S.* Typhimurium or with mutant bacteria deficient for *pipB2*, *sifA*, *sopD2*, *sseF*, *sseG* or *sseJ*. In parallel, cells were also infected with *sseL* and *steC* mutant bacteria, deficient for SPI-2 effectors with functions unconnected with membrane trafficking. By comparison to cells infected with wt *S.* Typhimurium (61 ± 3% of cells showing SCAMP3 tubules), there was a significant decrease in the frequency at which tubulated SCAMP3 appeared in cells infected with *pipB2* (27 ± 7%), *sopD2* (15 ± 3%), *sseF* (45 ± 5%) and *sseG* (38 ± 4%) mutant *S.* Typhimurium, while *sseJ* (57 ± 4%), *sseL* (53 ± 5%) and *steC* (58 ± 6%) mutants showed no significant defect (Fig. 7B). Bacteria deficient for SifA gradually lose their vacuolar membrane and by 14 h p.i. approximately 20% of intracellular bacteria remain inside vacuoles (Beuzón *et al*., 2000; Boucrot *et al*., 2005). Therefore, we only analysed *sifA* mutant bacteria that were surrounded by LAMP1 labelling (as marker of the SCV) and in these conditions we did not observe SCAMP3 tubulation (Fig. 7B). The deficiency in SCAMP3 tubulation displayed by *sopD2* and *pipB2* mutants was complemented when plasmids expressing SopD2 C-terminally tagged with double HA (SopD2-2HA; 50 ± 2% of infected cells showing SCAMP3 tubules) and PipB2-2HA (71 ± 1%) were introduced in the corresponding mutant strains, and the resulting bacteria were used to infect HeLa cells (Fig. 7B). The *sifA*, *sseF* and *sseG* mutants have been characterized as being non-polar by several laboratories (Hensel *et al*., 1998; Beuzón *et al*., 2000; Boucrot *et al*., 2005; Abrahams *et al*., 2006; Deiwick *et al*., 2006; Kuhle *et al*., 2006). We conclude that SifA, PipB2, SopD2, SseF and SseG mediate SCAMP3 tubulation. Therefore, SPI-2 effectors promote tubulation in HeLa cells not only by interfering with the endocytic pathway but also by manipulating post-Golgi trafficking.

### SCAMP3 and SCAMP2 are involved in kinesin-1-driven centrifugal trafficking of membranes from SCVs

We noted a distinct labelling pattern of SCAMP3 in cells infected with *pipB2* mutant *S.* Typhimurium (Fig. 8A). In 51 ± 6% of cells infected with the *pipB2* mutant, SCAMP3 accumulated around bacteria, and a similar but less dramatic pattern was observed in only 17 ± 5% of cells infected with wt *S.* Typhimurium (Fig. 8B). Analyses of single confocal z-sections suggested that the accumulation of SCAMP3 around *pipB2* mutant bacteria reflected its presence on the vacuolar membrane (Fig. S6). This suggested that PipB2 is involved in the outward trafficking of SCAMP3-enriched membranes from the SCV. Because PipB2 binds the centrifugal microtubule motor kinesin-1 (Henry *et al*., 2006), we determined if inhibition of kinesin-1 function would affect the formation of SCAMP3 tubules. HeLa cells were transfected with a DNA construct expressing GFP fused to the tetratricopeptide repeats (TPR) of the mouse kinesin light chain 2 (KLC2), a cargo-binding domain that, when overexpressed, has been shown to inhibit kinesin-1 (Rietdorf *et al*., 2001), or with a plasmid expressing GFP alone. After infection with wt *S.* Typhimurium for 14 h, we found that TPR overexpression inhibited, but did not abolish, the appearance of SCAMP3 tubules (Fig. 8C and Fig. S7A). These results indicate that SCAMP3 re-distributes from the SCV membrane into SCAMP3 tubules via kinesin-1 activity, and that other centrifugal motors are involved.

The results described above suggested that, in the absence of SCAMP3, kinesin-1-driven outward trafficking of tubules from the SCV might be impaired and this could result in a centrifugal force exerted on vacuoles containing wt bacteria, which could lead to their relocation to the periphery of infected cells. To test this hypothesis, we attempted to suppress the effect of the depletion of SCAMPs 2 and 3 on SCV positioning by inhibiting kinesin-1 function on the bacterial vacuoles. Overexpression of the TPR domain did suppress the SCV positioning phenotype displayed by cells depleted of SCAMPs 2 and 3 (Fig. 8D and Fig. S7B), and SCVs containing *pipB2* mutant bacteria were more resistant than wt vacuoles to depletion of the SCAMPs (Fig. 8E and Fig. S7C). Taken together, these results lead us to postulate that SCAMP3 and SCAMP2 contribute to kinesin-1-driven and PipB2-dependent outward trafficking of membranes from SCVs.

## Discussion

**Fig. 9:**
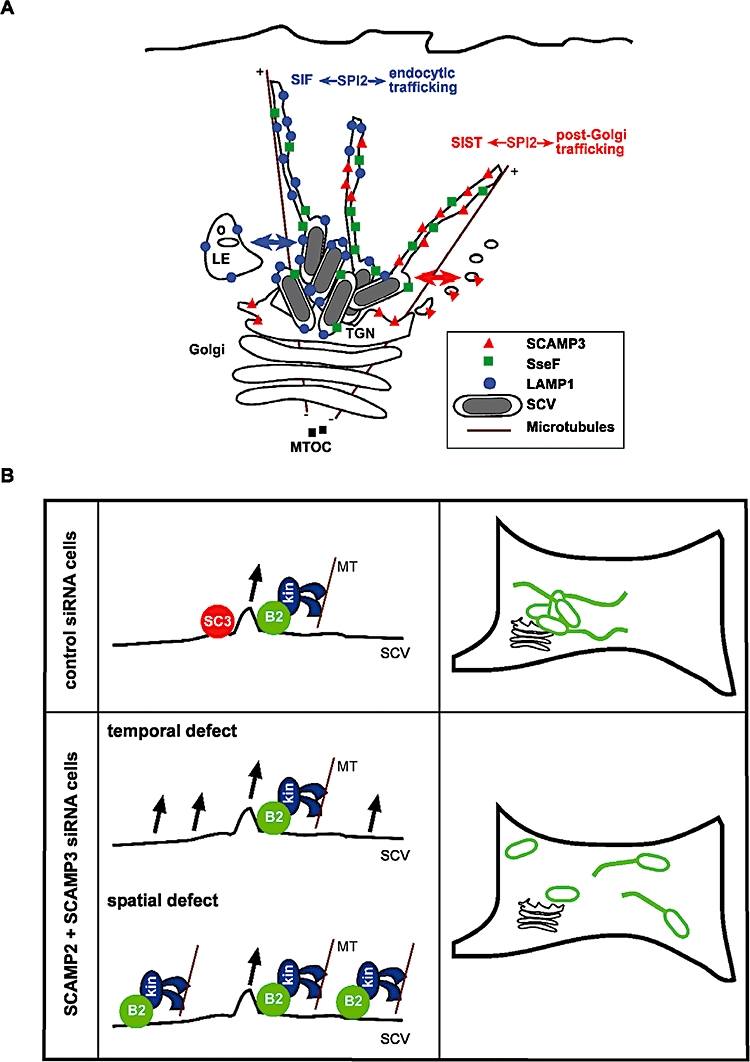
Model for the origins of SIFs/SISTs and for the function of SCAMP3 on the SCV. A. SPI-2 effectors mediate the formation of an extensive structures extending from the SCV that includes SIFs and SISTs in HeLa cells. SIFs were known to be decorated with proteins normally found in late endosomes (LEs), such as LAMP1, but the majority of SIFs also contains SCAMP3 (which concentrates on the TGN in uninfected cells). SISTs are marked by SCAMP3 and do not contain detectable levels of endosomal proteins. The appearance of SIFs with no SCAMP3 is rare. SPI-2 effectors, such as SseF, are present in both SIFs and SISTs. Therefore, SPI-2 effectors mediate the interaction between *Salmonella* and both the endocytic pathway and post-Golgi trafficking in HeLa cells, and this is reflected in the formation of SIFs and SISTs respectively. B. In the presence of SCAMP2 and SCAMP3 (control siRNA cells), PipB2-mediated kinesin-1 recruitment to sites of vesicle or tubule formation only occurs when a SCAMP-dependent microdomain allowing membrane fission and transport has been formed. In these conditions, SCVs remain essentially static while centrifugal transport of membranes occurs. SCAMP3 is constantly being incorporated into newly formed vesicles and tubules, resulting in the appearance of SCAMP3 tubules and eventually of SISTs. When SCAMPs are depleted (SC2+SC3 siRNA cells), the process of vesicle or tubule formation is affected such that kinesin-1 is recruited to sites of the vacuolar membrane that are not proficient for vesicle/tubule formation (spatial defect), or when such sites have not been formed yet (temporal defect). In these conditions, outward membrane trafficking still occurs, but the kinesin-1 centrifugal force is sensed by the SCVs, which then relocate from the Golgi region of the cell. B2, PipB2; kin, kinesin-1; MTs, microtubules; SC3, SCAMP3.

The most obvious manifestation of the interaction between *Salmonella* and the endocytic pathway is the reorganization in epithelial cells of late endosomal vesicles into dynamic tubular SIFs that extend from SCVs along microtubules, frequently for several micrometres (Garcia-del Portillo *et al*., 1993; Brumell *et al*., 2002; Drecktrah *et al*., 2008; Rajashekar *et al*., 2008). In this work, we show that the dynamic tubular network extending from the SCV in epithelial-derived cells also includes SCAMP3 and is much more extensive than previously thought, in that a significant proportion of tubules lack endosomal markers and are enriched in SCAMP3 (SISTs). As a major portion of SCAMP3 accumulates on the TGN at steady state (Castle and Castle, 2005; Fig. 2 and Fig. S3) and SCAMP3 tubulation is severely inhibited in the presence of BFA, we conclude that the *Salmonella*-induced tubular network is not only of endosomal origin but also results from interactions between SPI-2 effectors and the TGN or associated post-Golgi compartments (Fig. 9A).

Previous studies have suggested an interaction between *Salmonella* and the secretory pathway (Salcedo and Holden, 2003; Kuhle *et al*., 2006), but compelling evidence to support this has been lacking. Vesicles containing VSVG (a marker of the constitutive secretory pathway) are re-directed by SPI-2 effectors to accumulate in the vicinity of SCVs, but no clear evidence of fusion was detected (Kuhle *et al*., 2006). SCAMPs do not colocalize with VSVG-containing secretory vesicles (Castle and Castle, 2005), and SCAMP3 can be clearly detected on SCVs in living cells (Fig. 5). Therefore, considered together, the data indicate that *Salmonella* specifically intercepts and recruits membrane from a TGN-derived SCAMP3-containing pathway. This could be explained by direct or indirect mechanisms: SCAMP3-containing vesicles might be directly recruited from the TGN, or *Salmonella* could first re-direct SCAMP3 vesicles to late endosomes and then recruit SCAMP3 while interacting with those compartments. In both scenarios, TGN-derived membranes could fuse with the vacuolar membrane and/or with SCV-derived tubules.

The simultaneous appearance in the same cells of SIFs containing SCAMP3, of SISTs and, more rarely, of SIFs with no detectable SCAMP3 (Figs 3, 4 and 5C, and Fig. S5) shows that *Salmonella* sorts and mixes different membrane components, resulting in the appearance of these different tubules (Fig. 9A). It is noteworthy that SISTs are clear at 14 h p.i. but more difficult to detect at 8 h p.i. It is conceivable that over time, components of the secretory pathway within SIFs differentiate from endosomal tubules, resulting in the appearance of SISTs. It is also possible that SISTs might form independently at later time points. Therefore, it is unclear whether sorting occurs at the level of the SCV membrane or in tubules. In fixed cells, SISTs often appeared fragmented rather than as continuous tubules. This suggests that, as proposed for SIFs (Drecktrah *et al*., 2008), they are not preserved intact following fixation. Another possibility is that SCAMP3 and LAMP1 cluster independently in tubules and these represent intermediates in the membrane sorting process. Understanding the physiological significance and the mechanisms by which *Salmonella* mediates host cell membrane sorting is a major challenge of future research.

Remarkably, SCAMP3 tubulation and SIF formation require the same repertoire of SPI-2 T3SS effectors: PipB2, SifA, SopD2, SseF and SseG (Haraga *et al*., 2008; Fig. 7B). These effectors presumably comprise part of a common machinery underlying the process of SCV tubulation, regardless of the cellular origin of the membrane. Following its translocation across the SCV membrane, PipB2 binds directly to kinesin-1 and promotes its recruitment to the bacterial vacuoles (Henry *et al*., 2006). However, because of the action of SifA, kinesin-1 does not appear to be present on SCVs containing wt bacteria (Boucrot *et al*., 2005). SifA, anchored to the cytoplasmic face of the vacuolar membrane following isoprenoid modification of its C-terminus (Boucrot *et al*., 2003; Reinicke *et al*., 2005), binds to the PH domain of its host cell target SKIP (Boucrot *et al*., 2005), possibly displacing Rab9 in the process (Jackson *et al*., 2008). SifA-SKIP is then proposed to redirect kinesin-1 to generate SIFs that emanate from the SCV (Boucrot *et al*., 2005). Presumably this mechanism also underlies the formation of SCAMP3 tubules. However, inhibition of kinesin-1 only partially inhibits the formation of SCAMP3 tubules (Fig. 8C and Fig. S7A), indicating that other, as yet unknown, centrifugal motor(s) must be involved. The molecular functions of SopD2, SseF and SseG are not understood.

Different studies indicate that SCAMP isoforms associate with trafficking stations and certain vesicular carriers in post-Golgi recycling pathways and in the cell surface recycling system, but not in the constitutive secretory pathway (Castle and Castle, 2005). They are integral membrane proteins with cytoplasmic exposed N- and C- terminal regions; these might contain localization signals that determine their associations with specific pathways. Collectively, SCAMPs have been proposed to contribute to organizing membrane budding and fission sites (Castle and Castle, 2005). How then might SCAMPs 2 and 3 be involved in centrifugal trafficking of membranes from the SCV and be required for juxtanuclear positioning of bacterial vacuoles in HeLa cells SCAMPs 2 and 3 were not detected in coimmune precipitates with epitope-tagged versions of PipB2, SifA, SopD2, SseF or SseG, following the delivery of these effectors into the host cell by the SPI-2 T3SS (data not shown). Therefore, direct interaction with any of these SPI-2 effectors seems unlikely. Also, depletion of SCAMPs 2 and 3 did not noticeably affect the morphology of the Golgi network or the abundance of TGN proteins (data not shown). However, vesicle assembly must be synchronized with the recruitment of molecular motors driving transport on cytoskeletal tracks (Hehnly and Stamnes, 2007), and as mentioned above SCAMPs might organize membrane microdomains in such a way that they become a platform for sorting and budding (Castle and Castle, 2005). It is therefore conceivable that the action of SCAMP3 and SCAMP2 coordinates the formation of budding sites on the SCV with PipB2-mediated recruitment of kinesin-1 (Fig. 9B). In the absence of the SCAMPs, formation of these sites would be defective and the PipB2-kinesin-SifA-SKIP cascade could exert its effect directly on SCVs, leading to their peripheral relocation (Fig. 9B). Depletion of SCAMPs 2 and 3 did not result in a noticeable accumulation of kinesin-1 on the SCV (data not shown), and did not inhibit SIF formation (Fig. S2). Therefore, in the absence of the SCAMPs, outward trafficking of membranes from the SCV still occurs and relocation of the bacterial vacuoles probably results from a more subtle effect of kinesin-1 on the SCV. This would be consistent with the model, in which SCV positioning is determined by a balanced activity of cytoskeleton-based motors (Ramsden *et al*., 2007a; Wasylnka *et al*., 2008). The positioning defect of SCVs in cells depleted of SCAMPs 2 and 3 was suppressed by inhibition of kinesin-1 activity, suggesting that these two SCAMPs might function together on SCVs. Although SCAMP2 was not detected on SISTs or SIFs, it might be present on the SCV membrane or associated tubules, but at low levels.

Previous studies indicated that the inability of SCVs to localize to the Golgi region correlates with a deficiency in the intracellular replication of *S.* Typhimurium (Salcedo and Holden, 2003; Deiwick *et al*., 2006; Kuhle *et al*., 2006). However, depletion of SCAMPs 2 and 3 did not noticeably inhibit the intracellular replication of *Salmonella* (data not shown). Therefore, our data suggest that SCV localization to a juxtanuclear position is not essential for intracellular proliferation of *Salmonella*. Several intracellular pathogens have been shown to manipulate the host cell secretory pathway. Notably, *Legionella pneumophila*, *Brucella* spp., *Toxoplasma gondii* and several viruses interact with the endoplasmic reticulum to establish a protective and replicative niche and to manipulate host immune responses (Roy *et al*., 2006). Much less is known about the interaction between intracellular pathogens and post-Golgi trafficking. In addition to *Salmonella*, the only well-studied examples are *Chlamydia trachomatis* and *Chlamydophila pneumoniae*. Chlamydial vacuoles intercept a subset of Golgi-derived exocytic vesicles containing sphingomyelin and cholesterol, which are redirected to the chlamydial inclusion and are important for intracellular replication of the bacteria (Valdivia, 2008). The physiological significance of SCV tubulation is unknown. Nevertheless, *S.* Typhimurium strains deficient for each effector involved in the formation of SIFs and SCAMP3 tubules are attenuated in virulence in a mouse model of typhoid (Hensel *et al*., 1998; Beuzón *et al*., 2000; Brumell *et al*., 2003; Knodler *et al*., 2003). Therefore, it seems likely that the molecular mechanisms underlying the formation of these tubules are important for *Salmonella* virulence. It is plausible that interactions between *Salmonella* and SCAMP3 membranes could satisfy membrane or nutritional requirements. Another intriguing possibility is that the displacement of TGN-derived membranes into SCAMP3 tubules reflects an interference with host immune responses that involve trafficking to the plasma membrane, such as antigen presentation (Halici *et al*., 2008). Further work on the biogenesis and function of SCAMP3 tubules, and more specifically of SISTs, will improve our understanding of the intracellular biology of *Salmonella.* Furthermore, these studies may also help to shed light on the endogenous functions of SCAMPs, and their possible involvement in microtubule motor recruitment.

## Experimental procedures

### Bacterial strains, plasmids and DNA oligonucleotides

This work was done using wt *S.* Typhimurium NCTC 12023 (identical to ATCC 14208s) and its isogenic mutant derivatives. Detailed information on these bacterial strains is provided in Table S1. The plasmids and DNA oligonucleotides used in this work are detailed in Tables S2 and S3.

### Bacterial growth conditions, genetic procedures and DNA manipulations

*Escherichia coli* Top10 (Invitrogen) was used for construction and amplification of all plasmids. Bacteria were grown in Luria–Bertani (LB) medium (Invitrogen) supplemented when appropriate with ampicillin (50 μg ml^−1^), kanamycin (50 μg ml^−1^) and chloramphenicol (34 μg ml^−1^). The chromosomal deletion of *sopD2* in *S.* Typhimurium was performed by using the one-step gene-disruption method (Datsenko and Wanner, 2000).

Polymerase chain reactions (PCRs) were performed using *PfuUltra* (Stratagene) or the Expand High-Fidelity PCR System (Roche). Restriction enzymes and T4 DNA ligase were purchased from Invitrogen. Plasmid DNA was isolated using the GeneElute Plasmid Miniprep Kit (Sigma) or the QIAfilter Midi Kit (Qiagen). PCR products were purified using the QIAquick PCR purification Kit (Qiagen), and DNA fragments were purified from agarose gels using QIAquick Gel Extraction Kit (Qiagen). These DNA manipulations were performed according to the instructions of the different manufacturers and following standard procedures (Sambrook and Russel, 2001). DNA was introduced into *E. coli* or *S.* Typhimurium by electroporation using the GenePulser (Bio-Rad).

### Cell culture and bacterial infection

HeLa (93021013) cells were obtained from the European Collection of Cell Culture (Salisbury, UK). Cells were maintained in Dulbecco's modified Eagle medium (DMEM) high glucose (PAA laboratories) supplemented with 10% (v/v) foetal bovine serum (PAA laboratories), at 37°C in a humidified atmosphere containing 5% (v/v) in CO_2_. The cells were used up to 15 passages. Infection of HeLa cells with *S.* Typhimurium was performed as described (Beuzón *et al*., 2000).

### Reagents and drug treatments

All SCAMP-directed siRNA duplexes used in this work were obtained from Dharmacon and their sense sequences are provided in Table S3. The control siRNA was siCONTROL Non-Targeting siRNA#2 (Dharmacon).

Brefeldin A (SIGMA) and nocodazole (SIGMA) stock solutions were prepared in dimethyl sulfoxide (DMSO) and kept at −20°C. Working concentrations were 5 μg ml^−1^ for both BFA and nocodazole. To enable microtubule depolymerization, cells were incubated for 20 min at 4°C and then returned to 37°C in the presence of nocodazole as described (Ramsden *et al*., 2007b).

### Antibodies

For immunofluorescence microscopy, the rabbit SCAMP2 antibody (Wu and Castle, 1997) was used at 1:500; the rabbit SCAMP3 (Guo *et al*., 2002) antibody was used at 1:400; the rabbit anti-giantin antibody (Berkeley Antibody Company) was used at 1:600; the goat polyclonal anti-*Salmonella* antibody CSA-1 (Kirkegaard and Perry Laboratories) was used at 1:200; the mouse monoclonal antibodies anti-LAMP1 H4A3 and anti-CD63 H5C6, developed by J. T. August and J. E. K. Hildreth, obtained from the Developmental Studies Hybridoma Bank (DSHB) developed under the auspices of the NICHD and maintained by the University of Iowa (Department of Biological Sciences, Iowa, IA), were used at 1:1000 and at 1:2000 respectively; the mouse anti-LAMP2 monoclonal antibody CD3 was obtained from Dr Minoru Fukuda (The Burnam Institute, La Jolla Cancer Research Foundation, La Jolla, CA, USA) was used at 1:1000; the mouse anti-vATPase monoclonal antibody OSW2 was obtained from Dr Satoshi B. Sato (Kyoto University, Kyoto, Japan) and was used at 1:2000; the mouse anti-GM130 antibody (Transduction Laboratories) was used at 1:1000; the sheep anti-human TGN46 antibody (Serotec) was used at 1:100; the rat anti-HA 3F10 antibody (Roche) was used at 1:200; the mouse anti-TfR antibody (Zymed Laboratories) was used at 1:100; and the mouse anti-γ-tubulin (SIGMA; clone GTU-88) was used at 1:800 in methanol fixed cells. Secondary antibodies were obtained from Jackson Immunoresearch Laboratories: Cyanine 2 (Cy2)-, Cyanine 5 (Cy5)-, rhodamine red-X (RRX)- and aminomethylcoumarin acetate (AMCA)-conjugated donkey anti-mouse, anti-rabbit, anti-goat or anti-rat antibodies were used at 1:200, and Cy5- and RRX-conjugated anti-sheep antibodies were used at 1:100.

For immunoblotting, the antibodies were used at the following dilutions: the mouse anti-tubulin E7 antibody (DSHB) was used at 1:1000; the rabbit anti-SCAMP1 antibody (Hubbard *et al*., 2000) was used at 1:15 000; the rabbit anti-SCAMP2 antibody (Wu and Castle, 1997) was used at 1:2000; the rabbit anti-SCAMP3 antibody (Guo *et al*., 2002) was used at 1:4000; and the rabbit anti-SCAMP4 antibody (Hubbard *et al*., 2000) was used at 1:2000. Anti-mouse (IgG) and anti-rabbit (IgG) horseradish peroxidase secondary antibodies were obtained from GE Healthcare and used at 1:10 000.

### siRNA and DNA transfections

For siRNA transfections, 7.5 × 10^4^ HeLa cells were seeded in 24-well plates. About 24 h after plating, the cells were transfected with the different siRNAs, at 100 nM (siRNA pools and control) or at 25 nM (single siRNAs), by using 1.5 μl of DharmaFECT1 (Dharmacon), as detailed by the manufacturer. About 24 h after siRNA transfection, the cells were trypsinized, and were diluted 20-fold for seeding in 24-well plates, or diluted sixfold for seeding in 6-well plates (to assess the efficiency of siRNA knock-down by immunoblotting). About 72 h after siRNA transfection, the cells were infected with *S.* Typhimurium (see above) or processed for immunoblotting to assess the efficiency of siRNA-mediated depletion (see below).

HeLa cells were transfected with plasmid DNA by using the jetPEI reagent (Qbiogene), as detailed by the manufacturer. Cells were transfected with pGFP-KLC2-TPR 4 h before infection with *S.* Typhimurium, and with pEGFP-SCAMP3 and pLAMP1-HcRed 14–16 h before bacterial inoculation.

### Assessment of siRNA-mediated knock-downs

The cellular levels of SCAMPs after siRNA transfection were essentially assessed by imunoblotting. About 72 h after siRNA transfection, the cells were trypsinized, washed two times with phosphate-buffered saline, and re-suspended in SDS-PAGE sample loading buffer. The whole-cell extracts were separated in 15% (w/v) SDS-PAGE and transferred by electroblotting into PVDF membranes using Trans-Blot Semi-Dry (Bio-Rad). Developing was achieved by using ECL Plus (GE Healthcare) and the LAS1300 luminescent imager (Fujifilm). In terms of efficiency of depletion of the SCAMPs, similar results were obtained when the cells were collected 86 h after siRNA transfection (data not shown). It was also estimated by immunofluorescence microscopy that the efficiency of knock-down of the SCAMPs was 80–90% (data not shown).

### Immunofluorescence microscopy

Cells were fixed, permeabilized and incubated with antibodies as described (Beuzón *et al*., 2000). Labelled cells were analysed by using a fluorescence microscope (BX50; Olympus) or a laser scanning confocal microscope (LSM510; Carl Zeiss). With the exception of live-cell imaging analyses (see below), all images shown were obtained by confocal microscopy and represent combined projections of multiple 0.2–0.4 μm z-sections. The images were processed using Zeiss LSM Image Browser and Adobe Photoshop.

### Scoring of phenotypes by microscopy

To quantify SCV positioning, we surveyed cells infected with *Salmonella* expressing GFP and immunolabelled for giantin (as a Golgi marker) for association between a bacterial microcolony (a cluster of at least five bacterial vacuoles with overlapping fluorescence signals) and the Golgi. If no microcolony was detected, the cell was considered negative for Golgi association. If a microcolony was identified, the cell was scored positive for Golgi association only if (i) the bacterial fluorescence signal overlapped with the giantin labelling and (ii) the microcolony accounted for at least 80% of the total number of SCVs within the cell.

To quantify the appearance of SCAMP3 tubules, SIFs or SseF-HA tubules, the infected cells were considered positive for those structures if they showed at least one tubule-like structure longer than 2 μm (as judged from the length of a bacterial cell). Only infected cells showing a centrally located microcolony with approximately 15–25 fluorescent bacteria were considered. It was not possible to always fulfill this criterion when analysing cells infected with *ssaV* and *sifA* mutant *S.* Typhimurium, but cells infected with these bacteria did not show SCAMP3 tubules at all. We used LSCM to distinguish between SCAMP3-labelled tubules and LAMP1-marked SIFs, or between SCAMP3 tubules and SseF-HA tubules. Cells showing SCAMP3 tubules were first identified by looking in a channel that only allowed the visualization of SCAMP3 labelling; it was then evaluated whether the SCAMP3 tubules were distinct from SIFs or SseF-HA tubules. An infected cell was considered positive for the accumulation of SCAMP3 on the bacterial microcolony if all fluorescent bacteria within the microcolony appeared surrounded by SCAMP3 labelling.

At least 50 infected cells were scored blind in each experiment, and all experiments were repeated at least three times. All results are reported as mean ± standard error of the mean (SEM). Statistical analyses were performed with Prism 5 software (GraphPad) using one-way anova and Dunett *post hoc* analyses or two-tailed unpaired Student's *t*-test as indicated. Differences between data sets were considered significant if *P* < 0.05.

### Live-cell imaging

Live-cell imaging was performed as described before (Ramsden *et al*., 2007b). The cells were imaged on a Zeiss Axiovert 200 M microscope (Carl Zeiss). The images were recorded and processed using Volocity (Improvision).

## Supporting information

**Fig. S1.** The effect of different siRNAs on expression of SCAMP2 and SCAMP3 and on SCV positioning.

A. HeLa cells were transfected with the indicated siRNAs, and whole-cell extracts were subjected to immunoblotting using antibodies to β-tubulin, SCAMP2, and SCAMP3. The thick arrows indicate the single siRNAs used throughout this work. The thin arrows indicate the siRNAs used to further confirm in (B) the SCV positioning phenotypes after depletion of SCAMP2 and SCAMP3.

B. Quantification of SCV positioning phenotypes of HeLa cells transfected with the indicated siRNA and infected with wt S. Typhimurium for 14 h. In these experiments 70 ± 5% of the cells infected with wt bacteria that had been transfected with control siRNA showed a Golgi-associated bacterial microcolony. *P*-values were obtained one-way anova and Dunett *post hoc* analyses (***P* < 0.01; ****P* < 0.001) relative to control siRNA-treated cells. Values are mean ± SEM (*n* = 3).

**Fig. S2.** Depletion of SCAMPs 2 and SCAMP3 does not affect SCV migration to the MTOC and SIF formation.

A. Quantification of SCV migration to the MTOC. HeLa cells were transfected with the indicated siRNAs, infected with wt S. Typhimurium, and fixed with ice-cold methanol at 2 h p.i. The cells were immunolabelled for giantin, γ-tubulin, and *Salmonella*, and the distance of individual SCVs from the MTOC was determined as previously described (Ramsden *et al*., 2007b). Data are from three of the five siRNA experiments represented in Fig. 1.

B. Quantification of the appearance of SIFs. HeLa cells were transfected with the indicated siRNAs, and infected with wt S. Typhimurium expressing GFP for 14 h. The cells were fixed and immunolabelled for LAMP1. Data are from the same five experiments represented in Fig. 1.

C. Images illustrating that SIFs are still formed in SCAMP2 and SCAMP3 depleted cells. HeLa cells were infected and immunolabelled as described in (B). Arrowheads indicate SIFs. Scale bar, 5 μm. Statistical analyses were done by one-way anova and Dunett *post hoc* analyses (ns, not significant) relative to control cells.

**Fig. S3.** SCAMP3 concentrates on the TGN in uninfected HeLa cells. (A,B) HeLa cells were either treated with BFA for the indicated times or incubated in the presence of the solvent (DMSO) for 30 min. The cells were then fixed and immunolabelled as indicated. TGN46 is false coloured. Scale bars, 5 μm.

**Fig. S4.** SISTs can be identified at different times p.i. HeLa cells were infected with wt *S*. Typhimurium expressing GFP, fixed at the indicated times p.i. and immunolabelled as shown. LAMP1 and *Salmonella* are false coloured. Tubular structures are outlined in the insets. Arrows indicate the position of SISTs (tubules that do not overlap with LAMP1 labelling), and arrowheads indicate SCAMP3 tubules that colocalize with SIFs. All scale bars, 5 μm.

**Fig. S5.** All SCAMP3 tubules contain SseF-HA. Images illustrating that SCAMP3 tubules colocalize with SseF-HA tubules and can be distinguished from LAMP1-labelled SIFs. HeLa cells were infected with *sseF* mutant *S*. Typhimurium transformed with pSseF-HA, fixed at 14 h p.i., and immunolabelled as indicated. SCAMP3 tubules that colocalize with SIFs are shown in upper and middle panels (indicated by insets and arrowheads); SISTs (SCAMP3 tubules that lack late endosomal markers) are shown in middle and lower panels (indicated by insets and arrows). All scale bars, 5 μm.

**Fig. S6.** SCAMP3 localizes to the SCV membrane enclosing *pipB2* mutant bacteria. Consecutive confocal z-sections from Fig. 5A (lower panel) showing that SCAMP3 encircles *pipB2* mutant bacteria and partially colocalizes with LAMP1 on the vacuolar membrane. Scale bars, 5 μm.

**Fig. S7.** Involvement of SCAMPs 2 and 3 in kinesin-1- dependent movement of membranes from the SCV.

A. The images illustrate the effect of the expression of GFPKLC2- TPR on the appearance of SCAMP3 tubules. HeLa cells were transfected and infected as indicated in the legend of Fig. 6C. The cells were immunolabelled as shown. SCAMP3 and *Salmonella* are false coloured. SCAMP3 tubules are indicated by arrowheads.

B. Images illustrating that the SCV positioning defect displayed in SCAMP2- and SCAMP3-depleted cells infected with wt *S*. Typhimurium can be suppressed by expression of GFP-KLC2-TPR. HeLa cells were transfected with the indicated siRNAs and then transfected and infected as explained in the legend of Fig. 6D. The cells were immunolabelled for giantin and *Salmonella*.

C. Images showing that, by comparison to SCVs containing wt bacteria, vacuoles enclosing *pipB2* mutant *S*. Typhimurium are resistant to the effect of SCAMP2 and SCAMP3 depletion on SCV positioning. HeLa cells were siRNA-transfected as in (B), infected with the indicated *S*. Typhimurium strains expressing GFP, fixed at 14 h p.i., and immunolabelled to giantin. All scale bars, 5 μm.

**Table S1.** *S*. Typhimurium strains used in this work.

**Table S2.** Plasmids used in this work.

**Table S3.** DNA oligonucleotides and siRNAs used in this work.

**Movie S1.** Live-cell imaging analysis of SCAMP3 tubules. HeLa cells were transfected with pEGFP-SCAMP3, and infected with wt *S*. Typhimurium expressing DsRed. The cells were imaged at about 10 h p.i. every 10 s for 10 min. The Movie is 60 times accelerated relative to real time. Scale bar, 5 μm.

**Movie S2.** Live-cell imaging analysis of SCAMP3 tubules and SIFs. HeLa cells were transfected with pEGFP-SCAMP3 and pLAMP1-HcRed, and infected with wt *S*. Typhimurium expressing DsRed. The cells were imaged at about 10 h p.i. every 10 s for 10 min (of which only the first 4 min 50 s are shown). The Movie is 60 times accelerated relative to real time. Scale bar, 5 μm.

**Movie S3.** Live-cell imaging analysis of an SIST and an SIF that are adjacent but do not colocalize. Obtained from Movie S2 by cropping and magnification (2×) of the boxed region indicated in Fig. 3D. Scale bar, 5 μm.

**Movie S4.** Live-cell imaging analysis of EGFP-SCAMP3 on the SCV membrane. Obtained from Movie S1 by cropping and magnification (2×) of the region indicated in Fig. 3C. Scale bar, 5 μm.
