# Supplementary material for: SCAMP3 is a component of the Salmonella-induced tubular network and reveals an interaction between bacterial effectors and post-Golgi trafficking
Source: Cell Microbiol. 2009 Jun 3;11(8):1236–53. doi: 10.1111/j.1462-5822.2009.01329.x (PMC2730479; doi:10.1111/j.1462-5822.2009.01329.x)
Supplement: Supplementary file 1 [file cmi0011-1236-SD1.pdf]

SUPPLEMENTAL TABLES

Table S1. *S*. Typhimurium strains used in this work

Strain	Description	Reference
12023	wild-type	NTCC (Colindale, UK)
ssaV	*ssaV*::*aphT* (Km^r) in 12023 (HH119)	(Deiwick *et al.*, 1998)
pipB2	Δ*pipB2*::*km* (Km^r) in 12023 (DH217)	(Henry *et al.*, 2006)
sifA	*sifA*::mTn*5* (Km^r) in 12023 (P3H6)	(Beuzón *et al.*, 2000)
sopD2	Δ*sopD2* in 12023	This work
sseF	Δ*sseF*::*aphT* (Km^r) in 12023 (HH107)	(Hensel *et al.*, 1998)
sseG	Δ*sseG*::*aphT* (Km^r) in 12023 (HH108)	(Hensel *et al.*, 1998)
sseJ	Δ*sseJ* in 12023	(Lossi *et al.*, 2008)
sseL	Δ*sseL*::*km* (Km^r) in 12023	(Rytkonen *et al.*, 2007)
steC	Δ*steC*::*km* (Km^r) in 12023	(Poh *et al.*, 2008)

References

Beuzón, C.R., Meresse, S., Unsworth, K.E., Ruiz-Albert, J., Garvis, S., Waterman, S.R., *et al.* (2000). Salmonella maintains the integrity of its intracellular vacuole through the action of SifA. *Embo J* 19, 3235-3249.

Deiwick, J., Nikolaus, T., Shea, J.E., Gleeson, C., Holden, D.W. and Hensel, M. (1998). Mutations in Salmonella pathogenicity island 2 (SPI2) genes affecting transcription of SPI1 genes and resistance to antimicrobial agents. *J Bacteriol* 180, 4775-4780.

Henry, T., Couillault, C., Rockenfeller, P., Boucrot, E., Dumont, A., Schroeder, N., *et al.* (2006). The Salmonella effector protein PipB2 is a linker for kinesin-1. *Proc Natl Acad Sci U S A* 103, 13497-13502.

Hensel, M., Shea, J.E., Waterman, S.R., Mundy, R., Nikolaus, T., Banks, G., *et al.* (1998). Genes encoding putative effector proteins of the type III secretion system of Salmonella pathogenicity island 2 are required for bacterial virulence and proliferation in macrophages. *Mol Microbiol* 30, 163-174.

Lossi, N.S., Rolhion, N., Magee, A.I., Boyle, C. and Holden, D.W. (2008). The Salmonella SPI-2 effector SseJ exhibits eukaryotic activator-dependent phospholipase A and glycerophospholipid : cholesterol acyltransferase activity. *Microbiology* 154, 2680-2688.

Poh, J., Odendall, C., Spanos, A., Boyle, C., Liu, M., Freemont, P. and Holden, D.W. (2008). SteC is a Salmonella kinase required for SPI-2-dependent F-actin remodelling. *Cell Microbiol* 10, 20-30.

Rytkonen, A., Poh, J., Garmendia, J., Boyle, C., Thompson, A., Liu, M., *et al.* (2007). SseL, a Salmonella deubiquitinase required for macrophage killing and virulence. *Proc Natl Acad Sci U S A* 104, 3502-3507.

Table S2. Plasmids used in this work

Plasmid	Description/Construction	Reference
pKD3	Carries a chloramphenicol resistance gene cassette. Used to construct the *sopD2* mutant	(Datsenko *et al.*, 2000)
pKD46	Carries the λ Red recombinase. Used to construct the *sopD2* mutant	(Datsenko *et al.*, 2000)
pCP20	Carries the FLP recombinase. Used to construct the *sopD2* mutant	(Datsenko *et al.*, 2000)
pFPV25.1	Carries *gfpmut3A* under the control of the *rpsM* constitutive promoter. Introduced into *S*. Typhimurium strains for fluorescence visualization	(Valdivia *et al.*, 1997)
pDsRed	Carries a fluorescent-optimized DsRed protein under the control of an arabinose inducible promoter (P_{BAD}). Introduced into *S*. Typhimurium strains for fluorescence visualization	(Sorensen *et al.*, 2003)
pWSK29	Low-copy cloning vector	(Wang *et al.*, 1991)
pSseF-HA	pWSK29 derivative (p2643) carrying *sseF::HA* under the control of a SPI-2 promoter (P_{sseA})	(Kuhle *et al.*, 2004)
pPipB2-2HA	The *pipB2* coding region and 504 nucleotides upstream from the ATG codon was PCR-amplified from *S*. Typhimurium 12023 genomic DNA using primers #148 and #149, the PCR product was digested with *Eco*RI-*Bam*HI, and ligated into those sites of pWSK29. The *pipB2::2HA* insert is under the control of the *pipB2* promoter	This work
pSopD2-2HA	The *sopD2* coding region and 380 nucleotides upstream from the ATG codon was PCR-amplified from *S*. Typhimurium 12023 genomic DNA using primers #145 and #146, the PCR product was digested with *Eco*RI-*Bam*HI, and ligated into those sites of pWSK29. The *sopD2::2HA* insert is under the control of the *sopD2* promoter	This work
pEGFP-C1	Transfection vector	Clontech
pSCAMP3	pCMV-SPORT6 vector carrying human SCAMP3 cDNA (MGC-5222/IMAGE ID 2901296)	ATCC (Virginia, USA)
pEGFP-SCAMP3	Human SCAMP3 cDNA was PCR-amplified from pSCAMP3 using primers #128 and #129, the PCR product was digested with *Bgl*II-*Hin*dIII, and ligated into those sites of pEGFP-C1	This work
pLAMP1-HcRed	Human LAMP1 cDNA was ligated into the *Nhe*I and *Bam*HI sites of pHcRed1-N1 (Clontech)	Audrey Dumont and Stéphane Méresse
pGFP-KLC2-TPR	The GFP-KLC2-TPR insert is in the pCB6 transfection vector under control of the CMV promoter	(Rietdorf *et al.*, 2001)

References

Datsenko, K.A. and Wanner, B.L. (2000). One-step inactivation of chromosomal genes in Escherichia coli K-12 using PCR products. *Proc Natl Acad Sci U S A* 97, 6640-6645.

Kuhle, V., Jackel, D. and Hensel, M. (2004). Effector proteins encoded by Salmonella pathogenicity island 2 interfere with the microtubule cytoskeleton after translocation into host cells. *Traffic* 5, 356-370.

Rietdorf, J., Ploubidou, A., Reckmann, I., Holmstrom, A., Frischknecht, F., Zettl, M., *et al.* (2001). Kinesin-dependent movement on microtubules precedes actin-based motility of vaccinia virus. *Nat Cell Biol* 3, 992-1000.

Sorensen, M., Lippuner, C., Kaiser, T., Misslitz, A., Aebischer, T. and Bumann, D. (2003). Rapidly maturing red fluorescent protein variants with strongly enhanced brightness in bacteria. *FEBS Lett* 552, 110-114.

Valdivia, R.H. and Falkow, S. (1997). Fluorescence-based isolation of bacterial genes expressed within host cells. *Science* 277, 2007-2011.

Wang, R.F. and Kushner, S.R. (1991). Construction of versatile low-copy-number vectors for cloning, sequencing and gene expression in Escherichia coli. *Gene* 100, 195-199.

Table S3. DNA oligonucleotides and siRNAs used in this work

Primer / siRNA	Sequences[a]	Target sequence
DNA oligos		
DeltaSopD2_Fw	5’TTGCTTTCGCGGTAAATAATCAAGGGAGTTAT TATGCCAGGTGTAGGCTGGAGCTGCTTCG	*sopD2* and pKD3
DeltaSopD2_Rv	5’GGGGCCTTTTTAATGACTTTTTATATAAGCAT ATTGCGACCTATAGAATATCCTCCTTAG	*sopD2* and pKD3
PipB2_Fw_EcoRI (#148)	5’ATGCGAATTCACTCGATAGACTTATAACCG	Upstream from *pipB2*
PipB2_2xHA_Rv_BamHI (#149)	5’ATGCGGATCCCTAAGCATAATCAGGAACATCA TACGGATACGCATAGTCCGGCACATCATACGGA TAAATATTTTCACTATAAAATTCG	*pipB2::HA*
SopD2_Fw_EcoRI (#145)	5’ATGCGAATTCATCTATGCTGCATAACTCGC	Upstream from *sopD2*
SopD2_2xHA_Rv_BamHI (#146)	5’ATGCGGATCCTTAAGCATAATCAGGAACATCA TACGGATACGCATAGTCCGGCACATCATACGGA TATATAAGCATATTGCGACAACTCG	*sopD2::HA*
SCAMP3_Fw_BglII (#128)	5’GATCAGATCTATGGCTCAGAGCAGAGACGGC	*SCAMP3*
SCAMP3_Rv_HindIII (#129)	5’GATCAAGCTTCATCCCAGTCAGGGGTCACGG	*SCAMP3*
RNA duplexes		
SC1p	SC1#1: AAGAAAAGCCGCAGAAUUA<br>SC1#2: UGUCGGACCUUGUUUCUAU<br>SC1#3: GGGCAUUGGUGGUUGGAUU<br>SC1#4: CAGAAUGCUUUCAAGGGUA	*SCAMP1*
SC2p	SC2#1: GUAGCCAACUUGCAUGUGA<br>SC2#2: GGACAGCGGUUGGAUUGCA<br>SC2#3: UGUCUACACUGGAUAAUCA<br>SC2#4: GAACACUGUAGCCAACUUG	*SCAMP2*
SC3p	SC3#1: CCACAGAACCUAAGAACUA<br>SC3#2: GAAGGGCAACACAGCAGUA<br>SC3#3: GCAGAGGAGUUGGACCGAA<br>SC3#4: GGAAUUGUCAUGCUGAAAC	*SCAMP3*
SC4p	SC4#1: GCCGACAGCUCCUUUAAUU<br>SC4#2: CGAGGGAGGCCCAGUACAA<br>SC4#3: GCACAGACGGAGUGGAACA<br>SC4#4: CAGAAAAGGAGAACAACUU	*SCAMP4*
SC3#5	AACGGAUCCACUCCUUAUA	*SCAMP3*

[a] For the RNA duplexes only the sense sequence is shown.

SUPPLEMENTAL MOVIE LEGENDS

Movie S1. Live cell imaging analysis of SCAMP3 tubules. HeLa cells were transfected with pEGFP-SCAMP3, and infected with wt *S*. Typhimurium expressing DsRed. The cells were imaged at about 10 h p.i. every 10 s for 10 min. The Movie is 60 times accelerated relative to real time. Scale bar, 5 µm.

Movie S2. Live cell imaging analysis of SCAMP3 tubules and SIFs. HeLa cells were transfected with pEGFP-SCAMP3 and pLAMP1-HcRed, and infected with wt *S*. Typhimurium expressing DsRed. The cells were imaged at about 10 h p.i. every 10 s for 10 min (of which only the first 4 min 50 s are shown). The Movie is 60 times accelerated relative to real time. Scale bar, 5 µm.

Movie S3. Live cell imaging analysis of a SIST and a SIF that are adjacent but do not co-localize. Obtained from Movie S2 by cropping and magnification (2x) of the boxed region indicated in Figure 3D. Scale bar, 5 µm.

Movie S4. Live cell imaging analysis of EGFP-SCAMP3 on the SCV membrane. Obtained from Movie S1 by cropping and magnification (2x) of the region indicated in Figure 3C. Scale bar, 5 µm.

SUPPLEMENTAL FIGURE LEGENDS

Figure S1. The effect of different siRNAs on expression of SCAMP2 and SCAMP3 and on SCV positioning. (A) HeLa cells were transfected with the indicated siRNAs, and whole cell extracts were subjected to immunoblotting using antibodies to β-tubulin, SCAMP2, and SCAMP3. The thick arrows indicate the single siRNAs used throughout this work. The thin arrows indicate the siRNAs used to further confirm in (B) the SCV positioning phenotypes after depletion of SCAMP2 and SCAMP3. (B) Quantification of SCV positioning phenotypes of HeLa cells transfected with the indicated siRNA and infected with wt *S*. Typhimurium for 14 h. 70 ± 5 % of the cells infected with wt bacteria that had been transfected with control siRNA showed a Golgi-associated bacterial microcolony. P values were obtained one-way ANOVA and Dunett post-hoc analyses (** $P < 0.01$; *** $P < 0.001$) relative to control siRNA-treated cells. Values are mean ± SEM ($n = 3$).

Figure S2. Depletion of SCAMPs 2 and SCAMP3 does not affect SCV migration to the MTOC and SIF formation. (A) Quantification of SCV migration to the MTOC. HeLa cells were transfected with the indicated siRNAs, infected with wt S. Typhimurium, and fixed with ice-cold methanol at 2 h p.i. The cells were immunolabeled for giantin, γ-tubulin, and *Salmonella*, and the distance of individual SCVs from the MTOC was determined as previously described [Ramsden AE, Mota LJ, Munter S, Shorte SL, Holden DW (2007) The SPI-2 type III secretion system restricts motility of *Salmonella*-containing vacuoles. Cell Microbiol 9: 2517-2519]. Data are from 3 of the 5 siRNA experiments represented in Figure 1. (B) Quantification of the appearance of SIFs. HeLa cells were transfected with the indicated siRNAs, and infected with wt S. Typhimurium expressing GFP for 14 h. The cells were fixed and immunolabeled for LAMP1. Data are from the same 5 experiments

represented in Figure 1. (C) Images illustrating that SIFs are still formed in SCAMP2 and SCAMP3 depleted cells. HeLa cells were infected and immunolabelled as described in (B). Arrowheads indicate SIFs. Scale bar, 5 µm. Statistical analyses were done by one-way ANOVA and Dunett post-hoc analyses (ns, not significant) relative to control cells.

Figure S3. SCAMP3 concentrates on the TGN in uninfected HeLa cells. (A and B) HeLa cells were either treated with BFA for the indicated times or incubated in the presence of the solvent (DMSO) for 30 min. The cells were then fixed and immunolabeled as indicated. TGN46 is false colored. Scale bars, 5 µm.

Figure S4. SISTs can be identified at different times p.i. HeLa cells were infected with wt *S*. Typhimurium expressing GFP, fixed at the indicated times p.i. and immunolabeled as shown. LAMP1 and *Salmonella* are false colored. Tubular structures are outlined in the insets. Arrows indicate the position of SISTs (tubules that do not overlap with LAMP1 labeling), and arrowheads indicate SCAMP3 tubules that co-localize with SIFs. All scale bars, 5 µm.

Figure S5. All SCAMP3 tubules contain SseF-HA. Images illustrating that SCAMP3 tubules co-localize with SseF-HA tubules and can be distinguished from LAMP1-labeled SIFs. HeLa cells were infected with *sseF* mutant *S*. Typhimurium transformed with pSseF-HA, fixed at 14 h p.i., and immunolabeled as indicated. SCAMP3 tubules that co-localize with SIFs are shown in upper and middle panels (indicated by insets and arrowheads); SISTs (SCAMP3 tubules that lack late endosomal markers) are shown in middle and lower panels (indicated by insets and arrows). All scale bars, 5 µm.

Figure S6. SCAMP3 localizes to the SCV membrane enclosing *pipB2* mutant bacteria. Consecutive confocal z-sections from Figure 5A (lower panel) showing that SCAMP3 encircles *pipB2* mutant bacteria and partially co-localizes with LAMP1 on the vacuolar membrane. Scale bars, 5 μm.

Figure S7. Involvement of SCAMPs 2 and 3 in kinesin-1-dependent movement of membranes from the SCV. (A) The images illustrate the effect of the expression of GFP-KLC2-TPR on the appearance of SCAMP3 tubules. HeLa cells were transfected and infected as indicated in the legend of Figure 6C. The cells were immunolabeled as shown. SCAMP3 and *Salmonella* are false colored. SCAMP3 tubules are indicated by arrowheads. (B) Images illustrating that the SCV positioning defect displayed in SCAMP2- and SCAMP3-depleted cells infected with wt *S*. Typhimurium can be suppressed by expression of GFP-KLC2-TPR. HeLa cells were transfected with the indicated siRNAs and then transfected and infected as explained in the legend of Figure 6D. The cells were immunolabeled for giantin and *Salmonella*. (C) Images showing that, by comparison to SCVs containing wt bacteria, vacuoles enclosing *pipB2* mutant *S*. Typhimurium are resistant to the effect of SCAMP2 and SCAMP3 depletion on SCV positioning. HeLa cells were siRNA-transfected as in (B), infected with the indicated *S*. Typhimurium strains expressing GFP, fixed at 14 h p.i., and immunolabeled to giantin. All scale bars, 5 μm.

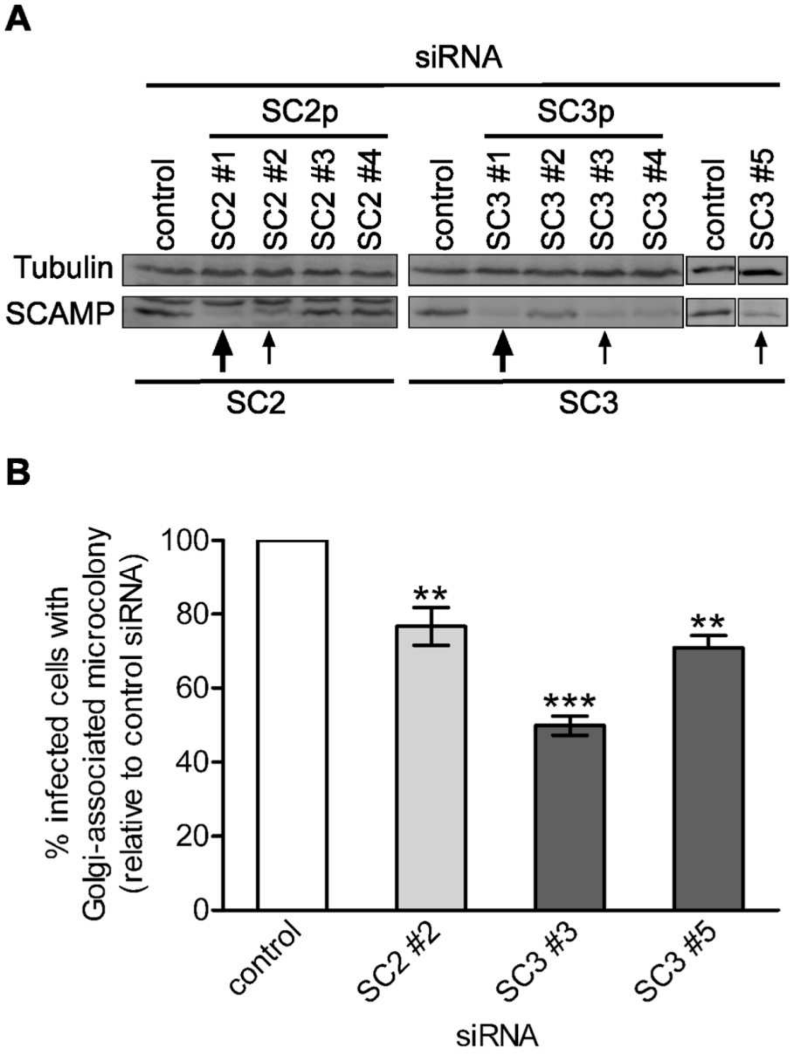

Figure S1, Mota *et al*

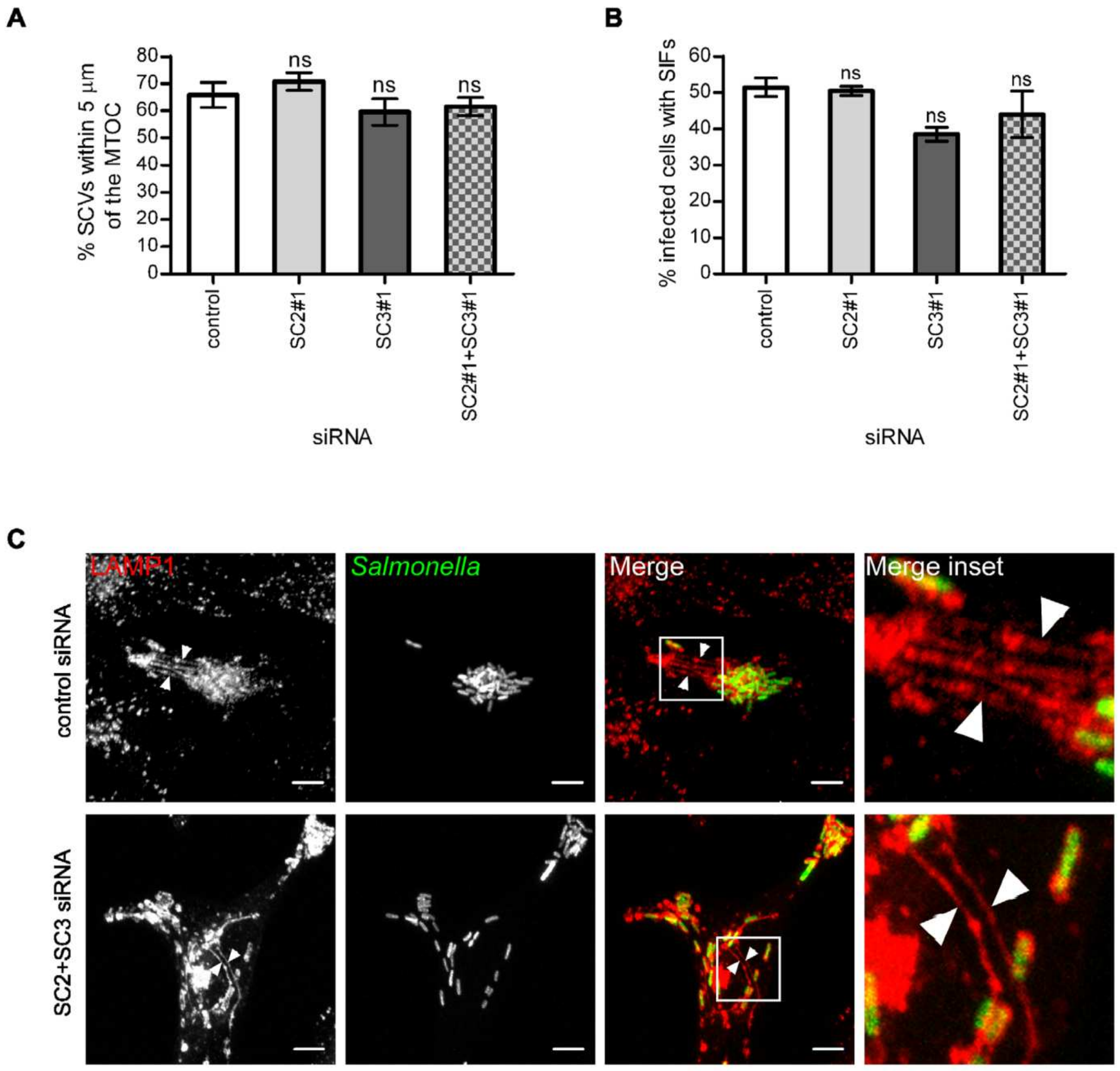

Figure S2, Mota *et al*

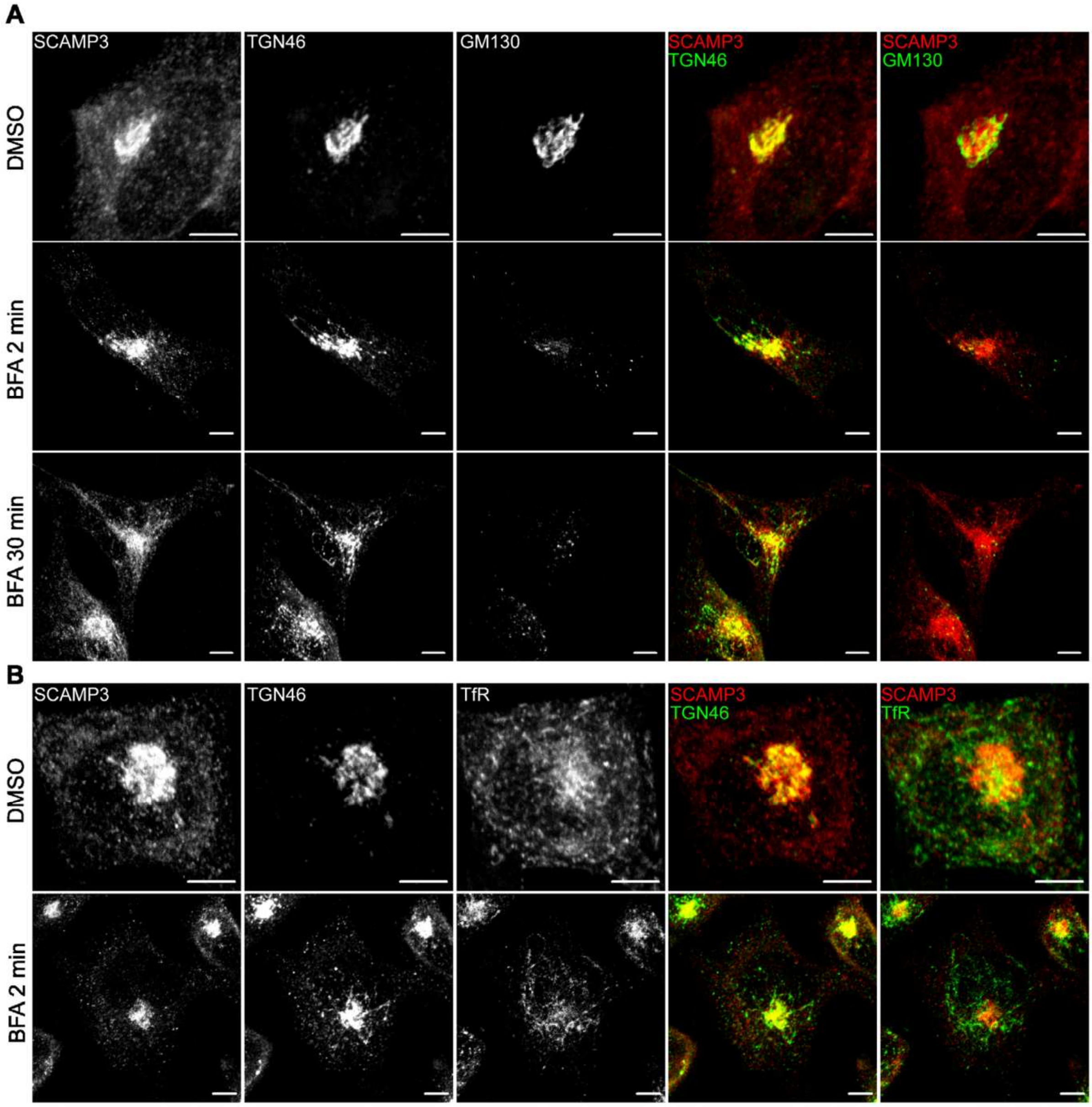

Figure S3, Mota *et al*

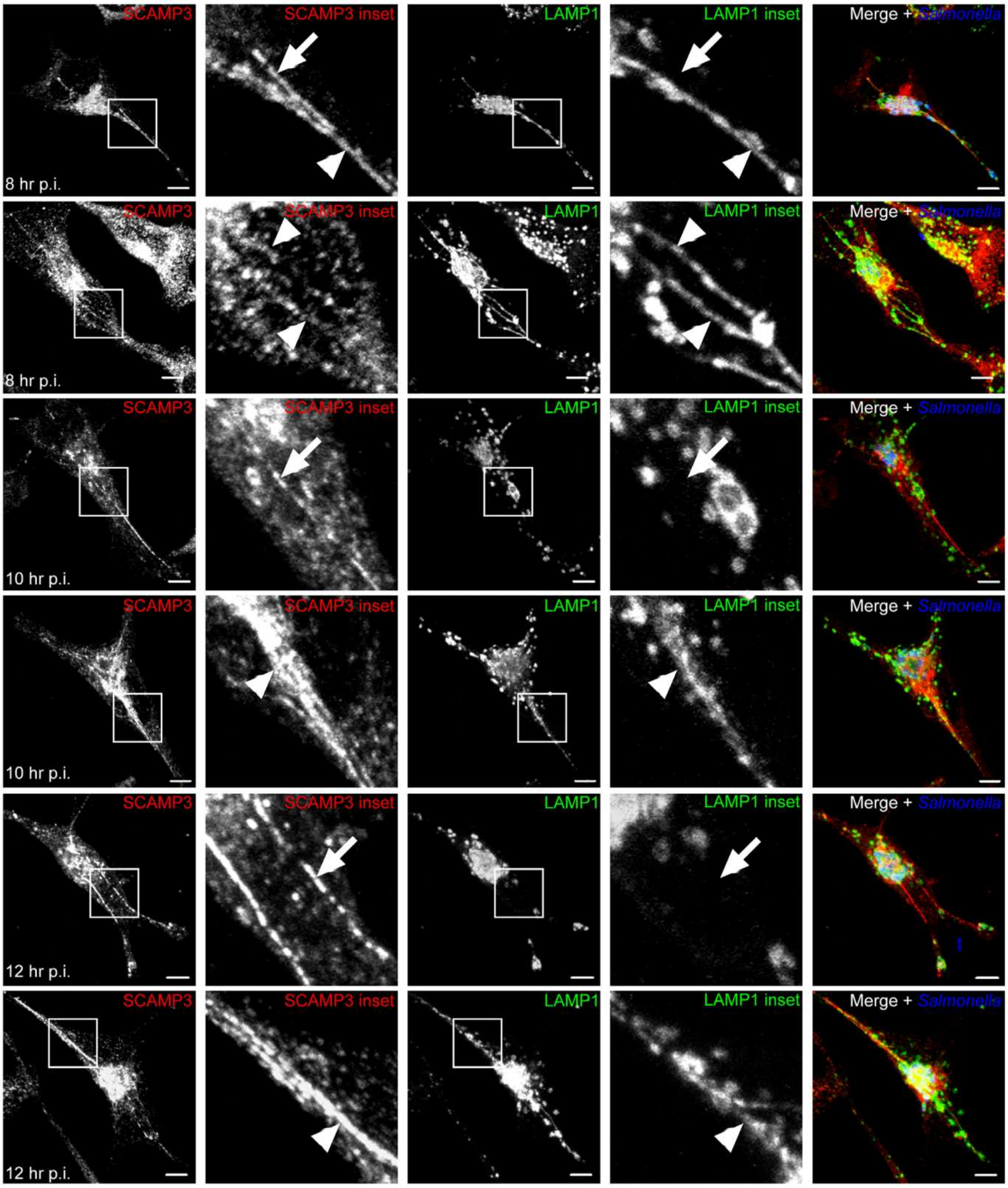

Figure S4, Mota *et al*

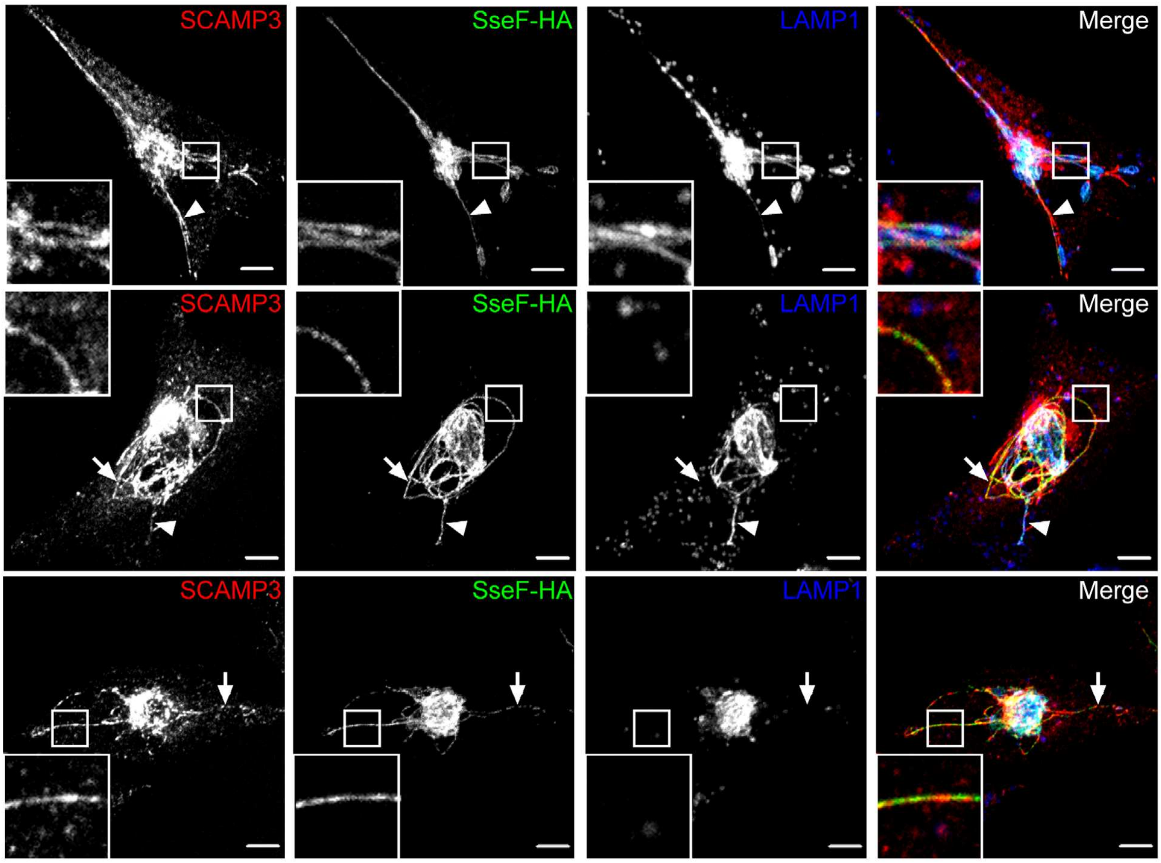

Figure S5, Mota *et al*

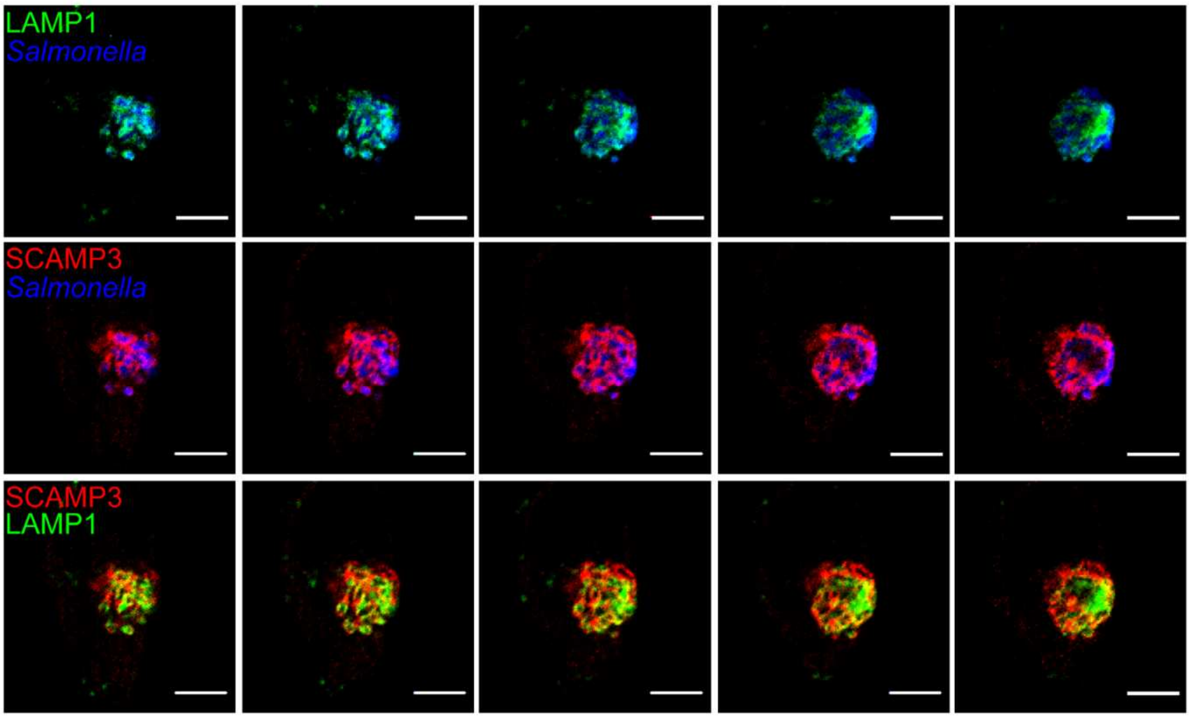

Figure S6, Mota *et al*

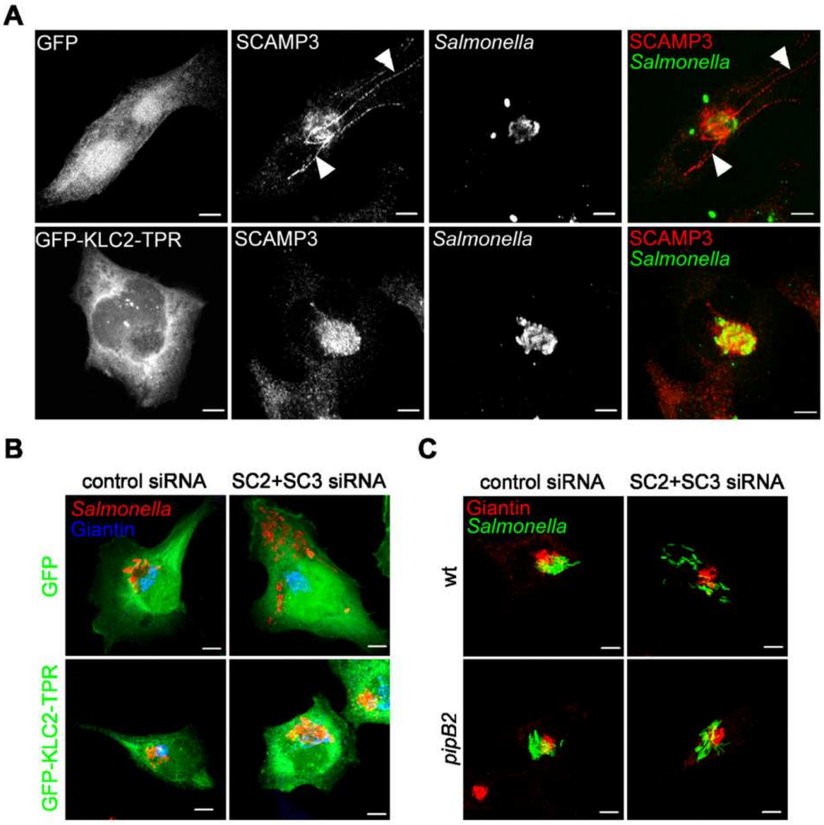

Figure S7, Mota *et al*
